# Simple Derivation of Spinal Motor Neurons from ESCs/iPSCs Using Sendai Virus Vectors

**DOI:** 10.1016/j.omtm.2016.12.007

**Published:** 2017-01-10

**Authors:** Kazuya Goto, Keiko Imamura, Kenichi Komatsu, Kohnosuke Mitani, Kazuhiro Aiba, Norio Nakatsuji, Makoto Inoue, Akihiro Kawata, Hirofumi Yamashita, Ryosuke Takahashi, Haruhisa Inoue

**Affiliations:** 1 Department of Neurology, Kyoto University Graduate School of Medicine, Kyoto 6068507, Japan; 2 Department of Cell Growth and Differentiation, Center for iPS Cell Research and Application (CiRA), Kyoto University, Kyoto 6068507, Japan; 3 Division of Gene Therapy and Genome Editing, Research Center for Genomic Medicine, Saitama Medical University, Saitama 3501241, Japan; 4 Institute for Integrated Cell-Material Sciences (WPI-iCeMS), Kyoto University, Kyoto 6068501, Japan; 5 DNAVEC Center, ID Pharma Co., Ltd., Tsukuba 3002611, Japan; 6 Department of Neurology, Tokyo Metropolitan Neurological Hospital, Tokyo 1830042, Japan

**Keywords:** motor neurons, Sendai virus, induced pluripotent stem cells, embryonic stem cells, iPSC, ESC, differentiation, direct conversion, transcription factor

## Abstract

Amyotrophic lateral sclerosis (ALS) is a progressive and fatal degenerative disorder of motor neurons (MNs). Embryonic stem cells (ESCs)/induced pluripotent stem cells (iPSCs) now help us to understand the pathomechanisms of ALS via disease modeling. Various methods to differentiate ESCs/iPSCs into MNs by the addition of signaling molecules have been reported. However, classical methods require multiple steps, and newer simple methods using the transduction of transcription factors run the risk of genomic integration of the vector genes. Heterogeneity of the expression levels of the transcription factors also remains an issue. Here we describe a novel approach for differentiating human and mouse ESCs/iPSCs into MNs using a single Sendai virus vector encoding three transcription factors, LIM/homeobox protein 3, neurogenin 2, and islet-1, which are integration free. This single-vector method, generating HB9-positive cells on day 2 from human iPSCs, increases the ratio of MNs to neurons compared to the use of three separate Sendai virus vectors. In addition, the MNs derived via this method from iPSCs of ALS patients and model mice display disease phenotypes. This simple approach significantly reduces the efforts required to generate MNs, and it provides a useful tool for disease modeling.

## Introduction

Amyotrophic lateral sclerosis (ALS), the most common and severe form of motor neuron diseases (MNDs), causes progressive muscle weakness and leads to death within several years. Vast amounts of findings concerning ALS have been reported, but the key mechanisms responsible for the disease are still not fully understood, hampering treatment. Consequently, the only FDA-approved drug, riluzole, was reported to prolong patient lifespan by just a few months.^1^ The establishment of induced pluripotent stem cells (iPSCs) offers a new approach to the study of MNDs and the discovery of new drugs.^2^, ^3^ In 2008, the first ALS patient iPSC-derived motor neurons (MNs) were generated.^4^ Since then, many ALS iPSC studies have been reported,^4^, ^5^, ^6^, ^7^, ^8^, ^9^, ^10^, ^11^, ^12^, ^13^, ^14^, ^15^, ^16^, ^17^, ^18^, ^19^, ^20^, ^21^, ^22^ and this technology is leading to new findings and therapeutic candidates for ALS.

MNs can be obtained from iPSCs, using signaling molecules such as retinoic acid (RA) and Sonic hedgehog (Shh) (Table S1).^4^, ^5^, ^12^, ^23^, ^24^, ^25^, ^26^, ^27^, ^28^, ^29^ These methods rely on developmental principles and require changing the combinations of signaling molecules at multiple steps, which is why some methods require more than 4 weeks to produce MNs. In contrast, Hester et al. reported a rapid differentiation method using adenoviral vectors that encode the transcription factors neurogenin 2 (Ngn2), islet-1 (Isl1), and LIM/homeobox protein 3 (Lhx3).^30^ These three transcription factors were transduced into neural progenitor cells, and MNs were obtained 11 days after the transduction. Son et al. reported that mouse and human fibroblasts were converted directly into MNs using seven and eight transcription factors, respectively, encoded by retrovirus vectors.^31^ In 2013, Mazzoni et al. generated doxycyclin-inducible mouse embryonic stem cell lines to obtain MNs^32^ (Table S2). Methods that rely on transcription factors are simple and rapid; but, when we use them for research on MNDs, we have to consider the possibility of genomic integration of the vector genes. Vector gene integration into host genomes contains the risk of influencing the behaviors of the transduced cells. Moreover, when we transduce several transcription factors separately, the transduction ratio of each transcription factor varies between the cells, and the heterogeneity of the cells may influence the experimental results. Therefore, we decided to focus on Sendai virus (SeV) vectors^33^, ^34^ (Table S3), which never integrate into host genomes with highly efficient transduction and expression levels of the transgene(s), and we designed a single SeV vector that encodes Lhx3, Ngn2, and Isl1 to produce more homogeneous MNs. Here we report that MNs can be induced from ESCs/iPSCs using a single SeV vector encoding a combination of transcription factors and that ALS iPSC-derived MNs exhibit disease phenotypes.

## Results

### Differentiation of Human iPSCs into MNs with Three Separate SeV Vectors

**Figure 1:**
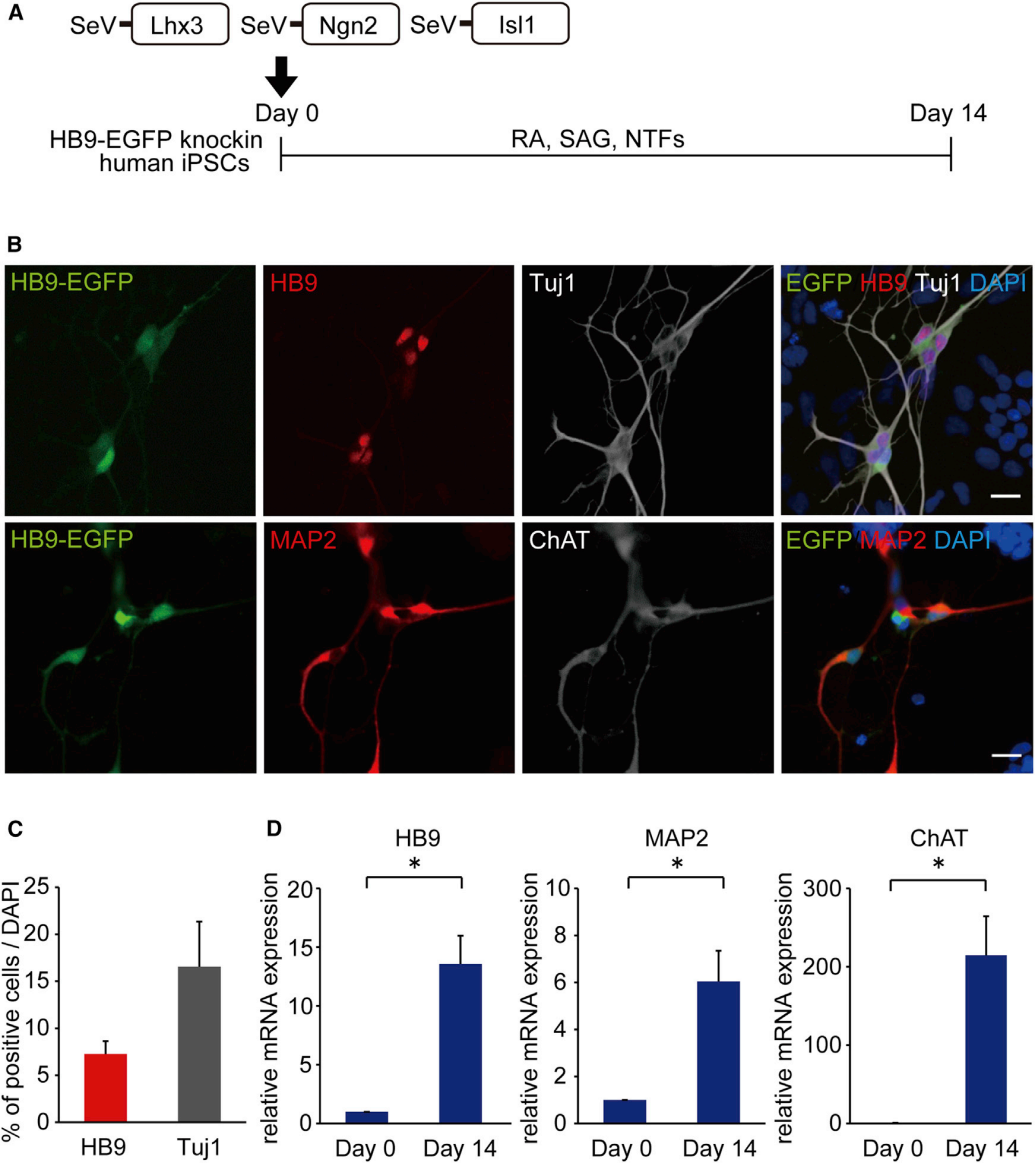
Differentiation of MNs with Three Separate Sendai Virus Vectors (A) Outline shows the experimental procedure to generate MNs using SeV-Lhx3, SeV-Ngn2, and SeV-Isl1 from HB9-EGFP knockin human iPSCs. (B) Immunofluorescence staining of iPSC-derived MNs for the MN markers HB9 and ChAT and for the neuronal markers Tuj1 and MAP2 is shown. Scale bars, 20 μm. (C) The percentages of HB9-positive and Tuj1-positive cells per total cells were 7.3% ± 1.4% and 16.6% ± 4.8% on day 14, respectively. Error bars are SEM (n = 6). (D) The qPCR analysis of differentiated cells on days 0 and 14 for MN markers (HB9 and ChAT) and neuronal marker (MAP2) is shown. Student’s t test was used for statistical comparison (*p < 0.05). Error bars are SEM (n = 3).

First, we differentiated human iPSCs into MNs as described in Figure 1A. To detect MNs easily, we used HB9-EGFP knockin human iPSCs.^35^ On day 0, iPSCs were seeded on Matrigel-coated dishes and the medium was changed from ESC medium to neurobasal medium with N2 and B27 supplements. RA, smoothened agonist (SAG), and neurotrophic factors (NTFs) also were added from day 0. For the differentiation to MNs, three separate vectors, SeV18+Lhx3/TS7ΔF (SeV-L), SeV18+Ngn2/TS7ΔF (SeV-N), and SeV18+Isl1/TS7ΔF (SeV-I) were transduced into human iPSCs. To test the transduction efficiency of the SeV vectors, we transduced SeV18+EGFP/TS7ΔF (SeV-EGFP) into control iPSCs at the multiplicity of infections (MOIs) of 1, 3, 10, 30, and 100. We observed dose-dependent increases of EGFP-positive cells on day 2. However, the ratios of EGFP-positive cells on day 4 compared to day 2 decreased at MOIs of 30–100 (Figure S1). Thus, we chose an MOI of less than 30. On day 14, we observed HB9-positive neurons and Tuj1-positive neurons, and 7.3% ± 1.4% and 16.6% ± 4.8% of total cells were positive for HB9 and Tuj1, respectively (Figures 1B and 1C). The qPCR analysis showed increased expression levels of HB9, ChAT, and MAP2 (Figure 1D). We also confirmed via immunocytochemistry and qPCR analysis that MNs can be generated from human ESCs with these SeV vectors (Figure S2).

To determine which combination of Lhx3, Ngn2, and Isl1 best produces MNs from iPSCs, we transduced one to three of SeV-L, SeV-N, and SeV-I into human iPSCs, and we evaluated Tuj1 and HB9 expressions by immunocytochemistry. The combination of all three factors and the combination of SeV-L and SeV-N produced both Tuj1- and HB9-positive neurons. The percentage of MNs per neurons was 43.9% ± 6.6% with all three factors and 18.2% ± 1.1% with Lhx3 and Ngn2 (Figure S3). We, therefore, decided to use all three factors for the differentiation of MNs.

### Differentiation to MNs by Lhx3, Ngn2, and Isl1 in a Single SeV Vector and Time-Lapse Imaging

**Figure 2:**
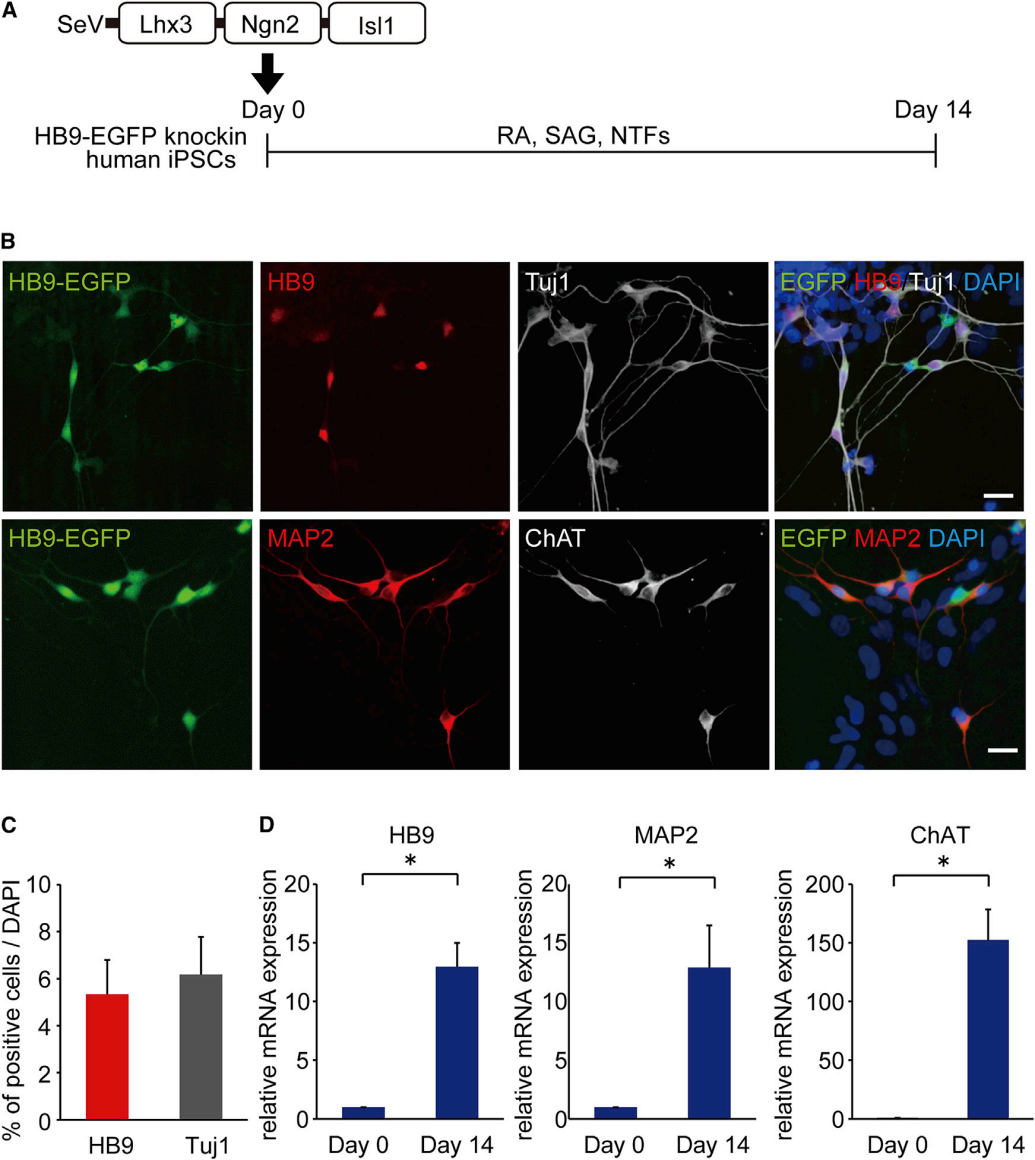
Differentiation of MNs with a Single SeV Vector Encoding Lhx3, Ngn2, and Isl1 (A) Outline shows the experimental procedure to generate MNs from HB9-EGFP knockin human iPSCs using a single vector, SeV-Lhx3-Ngn2-Isl1 (SeV-L-N-I). (B) Immunofluorescence staining of differentiated cells for HB9, Tuj1, MAP2, and ChAT is shown. Scale bars, 20 μm. (C) The percentages of HB9-positive and Tuj1-positive cells per total cells on day 14 were 5.3% ± 1.5% and 6.2% ± 1.6%, respectively. Error bars are SEM (n = 3). (D) The qPCR analysis of the differentiated cells on days 0 and 14 for HB9, ChAT, and MAP2 is shown. Student’s t test was used for statistical comparison (*p < 0.05). Error bars are SEM (n = 3).

To increase the percentage of MNs per neurons, we designed a single SeV vector encoding Lhx3, Ngn2, and Isl1 (SeV-L-N-I). Each transgene was connected with the transcription termination (E), trinucleotide intergenic (I), and transcription restart sequence (S) of the Sendai virus (EIS sequence). We examined the differentiation of MNs using this vector (Figure 2A). On day 14, we observed both HB9-positive neurons and ChAT-positive neurons, and 6.2% ± 1.6% of the total cells were neurons and 5.3% ± 1.5% were MNs. The percentage of MNs per neurons was 85.6% ± 1.7% (Figures 2B and 2C). We confirmed no MNs were obtained without SeV-L-N-I or with SeV-EGFP vector only using the current protocol. Without RA and SAG, HB9-positive cells were 48.5% ± 1.5% of neurons (Figures S4A–S4C). We also analyzed populations other than neurons (Figure S4D). The qPCR analysis showed increased expression levels of HB9, ChAT, and MAP2 (Figure 2D). By electrophysiological patch-clamp analysis, we observed the action potentials of generated MNs when co-cultured with primary astrocytes (Figure S4E). When MNs were co-cultured with human myocytes differentiated from a human myogenic cell line, Hu5/E18, the formation of neuromuscular junctions was confirmed by co-localization of HB9-EGFP-positive neurites with α-bungarotoxin-stained acetylcholine receptors (Figure S4F).

To capture when HB9-positive cells emerge, we conducted time-lapse imaging analysis using HB9-EGFP knockin iPSCs. Time-lapse imaging of EGFP was started on day 1, and EGFP-positive cells were observed on day 2. The number of EGFP-positive cells gradually increased, but some of them disappeared as time passed. On day 3, neuron-like morphology was observed (Movie S1).

### Differentiation to MNs by a Single SeV Vector Encoding Lhx3, Ngn2, Isl1, and EGFP

**Figure 3:**
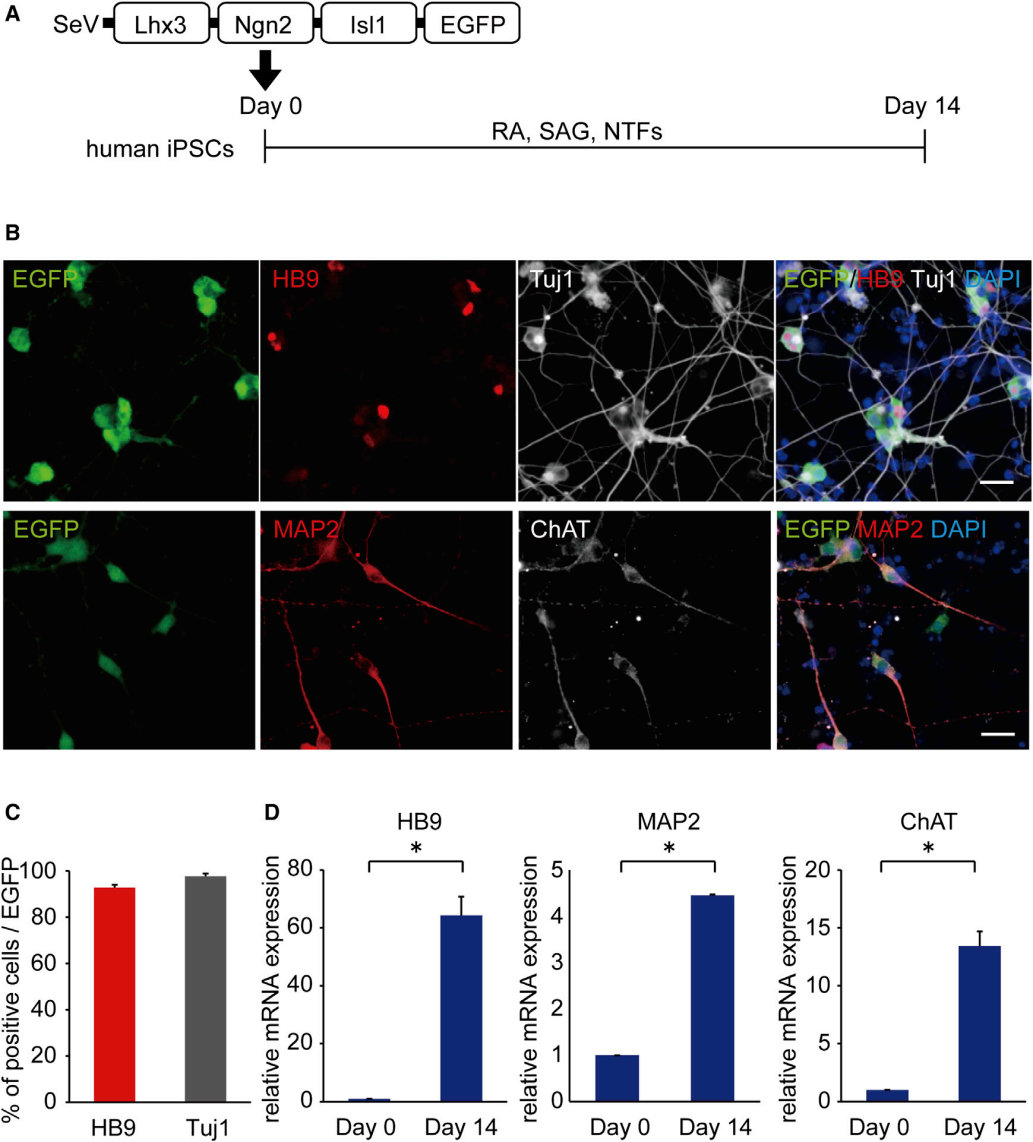
MN Differentiation Using a Single SeV Vector Encoding Lhx3, Ngn2, Isl1, and EGFP (A) Outline shows the experimental procedure to generate MNs using SeV-L-N-I-EGFP from iPSCs. (B) Immunostaining for HB9, Tuj1, MAP2, and ChAT on day 14 is shown. Scale bars, 20 μm. (C) Differentiation efficiency of MNs in SeV-infected cells is shown. The percentages of HB9-positive and Tuj1-positive cells per EGFP-positive cells were 92.8% ± 1.2% and 97.7% ± 1.2% on day 14, respectively. Error bars are SEM (n = 3). (D) The qPCR analysis for HB9, MAP2, and ChAT is shown. Student’s t test was used for statistical comparison (*p < 0.05). Error bars are SEM (n = 3).

**Figure 4:**
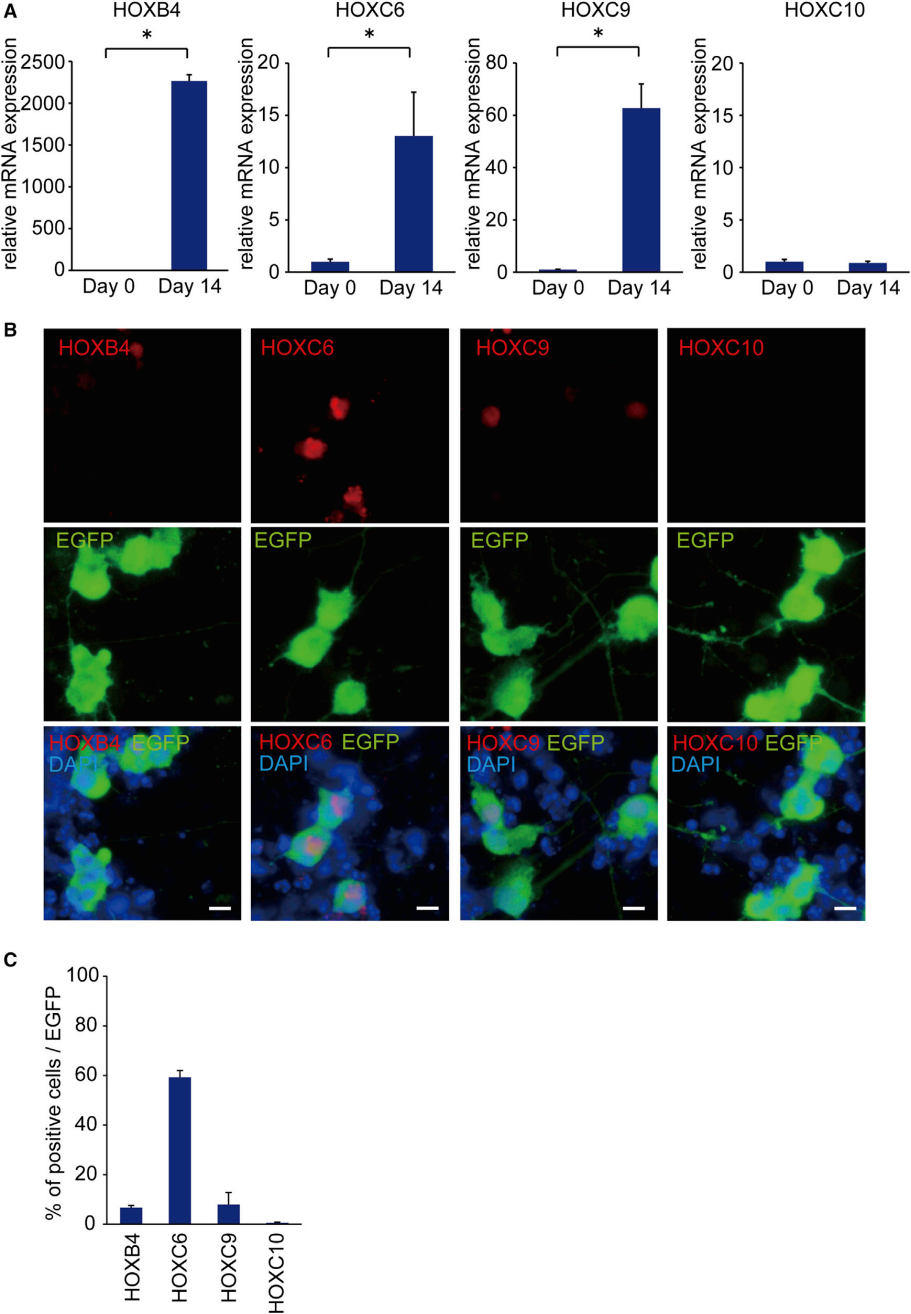
Characterization of MN Gene Expression Profile by a Single SeV Vector Encoding Lhx3, Ngn2, Isl1, and EGFP (A) The qPCR analysis for HOXB4, HOXC6, HOXC9, and HOXC10 is shown. Student’s t test was used for statistical comparison (*p < 0.05). Error bars are SEM (n = 3). (B) Immunostaining for HOXB4, HOXC6, HOXC9, and HOXC10 in MNs made by the SeV-L-N-I-EGFP vector is shown. Scale bars, 10 μm. (C) The percentage of HOX gene-positive cells per EGFP-positive cells is shown. Error bars are SEM (n = 3).

**Figure 5:**
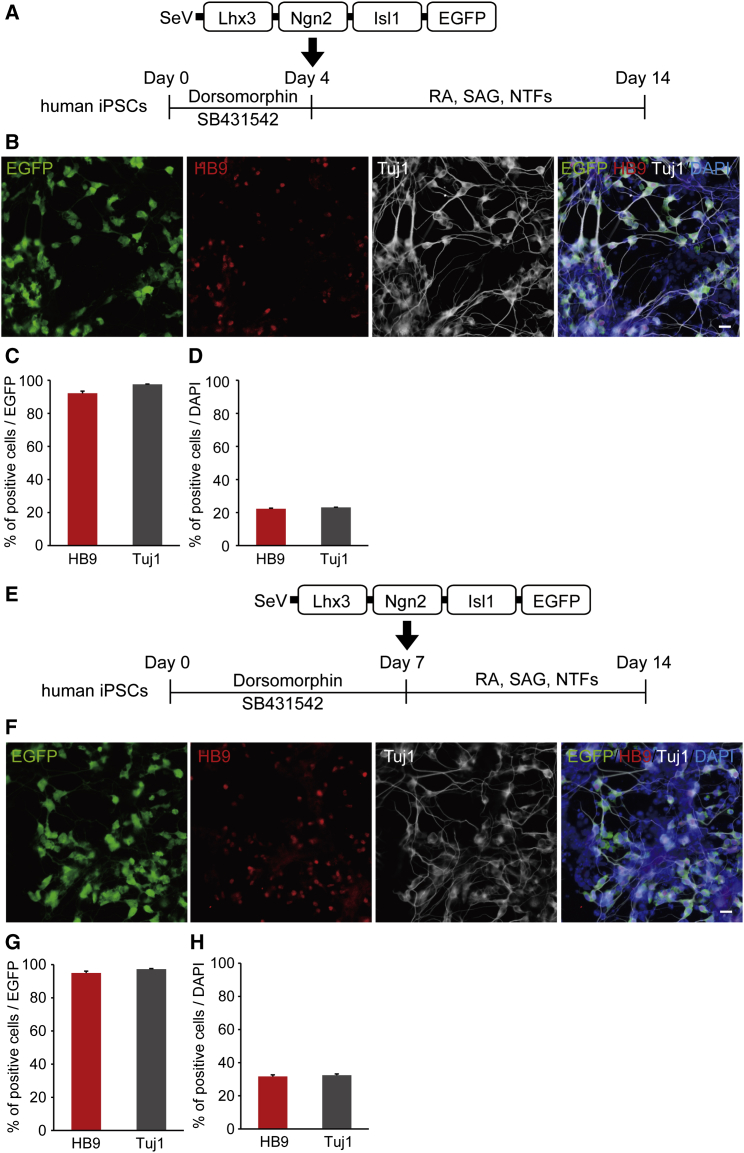
Differentiation of MNs from Neural Lineage Cells with a Single SeV Vector Encoding Lhx3, Ngn2, Isl1, and EGFP (A) Outline shows the experimental procedure to generate MNs using SeV-L-N-I-EGFP from neural lineage cells treated with dorsomorhpin and SB431542 for 4 days. (B) Immunostaining for HB9 and Tuj1 on day 14 is shown. Scale bar, 20 μm. (C) Differentiation efficiency of MNs in SeV-infected cells is shown. The percentages of HB9-positive and Tuj1-positive cells per EGFP-positive cells were 94.0% ± 1.3% and 97.6% ± 0.2% on day 14, respectively. Error bars are SEM (n = 3). (D) The percentages of HB9-positive and Tuj1-positive cells per total cells were 22.2% ± 0.4% and 23.1% ± 0.1% on day 14, respectively. Error bars are SEM (n = 3). (E) Outline shows the experimental procedure to generate MNs using SeV-L-N-I-EGFP from neural lineage cells treated with dorsomorhpin and SB431542 for 7 days. (F) Immunostaining for HB9 and Tuj1 on day 14 is shown. Scale bar, 20 μm. (G) Differentiation efficiency to MNs of SeV-infected cells is shown. The percentages of HB9-positive and Tuj1-positive cells per EGFP-positive cells were 95.0% ± 1.1% and 97.3% ± 0.3% on day 14, respectively. Error bars are SEM (n = 3). (H) The percentages of HB9-positive and Tuj1-positive cells per total cells were 31.6% ± 1.0% and 32.4% ± 0.8% on day 14, respectively. Error bars are SEM (n = 3).

Since the efficiency for the differentiation of neural lineage appeared low based on the total number of cells, to investigate the MN and neuron differentiation efficiency in SeV-infected cells, we designed an SeV-L-N-I-EGFP vector, which could label SeV-infected cells, and we transduced the three factors into iPSCs (Figure 3A). We found that >90% of SeV-L-N-I-EGFP-infected cells had differentiated into MNs and neurons (Figures 3B and 3C). The qPCR analysis showed increased expression levels of HB9, ChAT, and MAP2 (Figure 3D). Then, to evaluate the terminal subtypes of MNs along the rostrocaudal axis of the spinal cord, we examined the expression of HOX genes by qPCR and immunostaining.^36^ We observed increased mRNA expression of HOXB4, HOXC6, and HOXC9 on day 14, but the expression change of HOXC10 was not significant. Immunostaining showed that HOXC6-positive cells were about 60% of EGFP-positive cells (Figure 4). To analyze the efficiency for the differentiation to MNs from neural lineage cells, we transduced SeV-L-N-I-EGFP after treatment with dorsomorphin and SB431542 for 4 or 7 days (Figure 5). These results showed that the differentiation to MNs from neural lineage cells increased the number of HB9-positive cells compared to that from iPSCs.

### SOD1-ALS and TDP-43-ALS MNs, Differentiated by a Single SeV Vector Encoding Lhx3, Ngn2, and Isl1, Exhibit Disease-Specific Phenotypes

**Figure 6:**
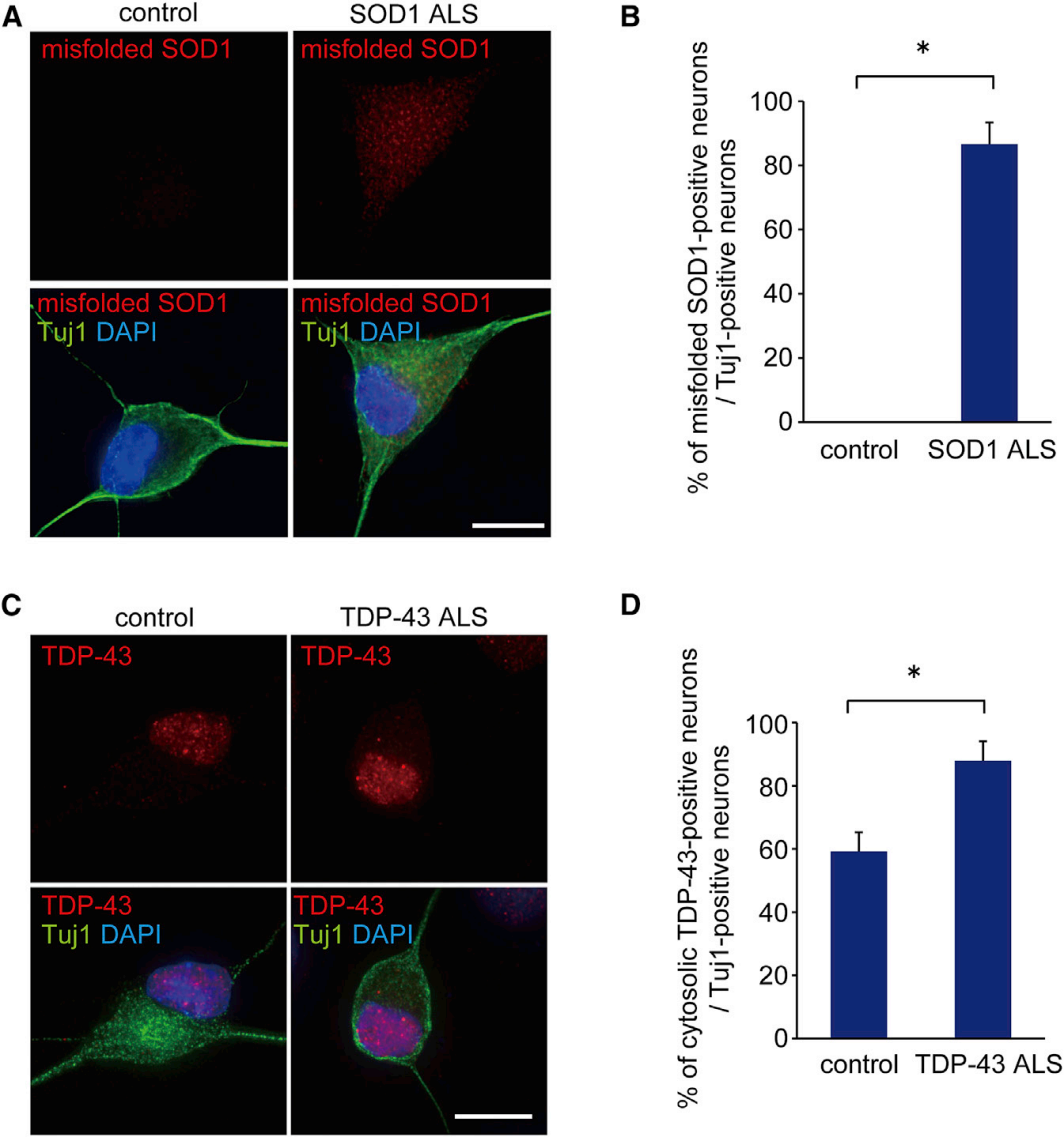
Phenotypes of SOD1-ALS and TDP-43-ALS iPSC-Derived Neurons by a Single SeV Vector Encoding Lhx3, Ngn2, and Isl1 (A) Immunostaining of misfolded SOD1 in control and SOD1-ALS iPSC-derived neurons is shown. Scale bar, 10 μm. (B) The percentages of misfolded SOD1-positive neurons in control and SOD1-ALS iPSC-derived neurons are shown. Student’s t test was used for statistical comparison (*p < 0.05). Error bars are SEM (n = 3). (C) Immunostaining of TDP-43 in control and TDP-43-ALS iPSC-derived neurons is shown. Scale bar, 10 μm. (D) The percentages of TDP-43 aggregation-positive neurons in control and TDP-43-ALS iPSC-derived neurons are shown. Student’s t test was used for statistical comparison (*p < 0.05). Error bars are SEM (n = 3).

To confirm that our method is applicable to the research of MNDs, we generated human iPSCs from the fibroblasts of a familial ALS patient with mutant superoxide dismutase 1 (SOD1 ALS) by transducing the four transcription factors Oct3/4, Sox2, Klf4, and c-Myc, as previously reported^3^, ^7^ (Table S4). The iPSCs were examined immunocytochemically for the ESC markers SSEA4 and NANOG (Figure S5A), and they were confirmed to retain the SOD1 gene mutation (Figure S5B). The generation of another familial ALS patients (mutant TAR DNA-binding protein, 43 kDa [TDP-43]-mediated ALS [TDP-43 ALS]), as well as control-derived iPSC lines, was reported previously.^7^ When we differentiated human ALS iPSCs into MNs using SeV-L-N-I (Figures S5C and S5D), SOD1-ALS iPSC-derived neurons presented an accumulation of misfolded SOD1 (Figures 6A and 6B), and TDP-43-ALS iPSC-derived neurons exhibited cytosolic TDP-43 aggregation (Figures 6C and 6D). These cellular phenotypes were not specific to MNs (Figures S5E and S5F).

Next, we generated iPSCs from embryonic fibroblasts of ALS model mice carrying mutant SOD1^37^ or mutant TDP-43^38^ or from littermate controls by transducing Oct3/4, Sox2, Klf4, and c-Myc, as previously reported^39^, ^40^ (Figure S6A; Table S5). We differentiated the iPSCs into MNs to examine their phenotypes via immunocytochemistry (Figures S6B and S6C). MNs derived from mouse SOD1-ALS iPSCs were positive for misfolded SOD1, while those derived from mouse control iPSCs were negative (Figure S6D). MNs derived from mouse TDP-43-ALS iPSCs did not display the cytosolic aggregates of TDP-43 (Figure S6E), which is consistent with a report on TDP-43-transgenic mice.^41^

## Discussion

Along with the development of stem cell technology, stem cell-derived MNs have been utilized for modeling MNDs in vitro. However, the heterogeneity of these MN populations presents a potential issue for disease modeling and analysis. To obtain more homogeneous MNs, we used a single SeV vector that encodes three transcription factors. SeV, known as murine parainfluenza virus type 1, is a negative sense, single-stranded RNA virus of the family *Paramyxoviridae*. SeV vectors are cytoplasmic RNA vectors that do not integrate into host genomes.^42^ They can be transduced into both dividing and non-dividing cells, and short-term exposure is enough for efficient transduction.^43^ SeV vectors can accommodate up to 5 kb of insertion.

The present study demonstrated that the ratio of MNs to neurons was higher when using a single SeV vector in comparison with three different SeV vectors for the transduction of Lhx3, Ngn2, and Isl1. The differentiation of neural lineage cells to MNs increases the percentage of HB9-positive cells per total cells compared to that of iPSCs. However, this method requires the dissociation and passage of cells and the change of compounds. On the other hand, the direct addition of a single vector to iPSCs is a very simple method, and the rapid differentiation of MNs is beneficial for research application. Moreover, immunocytochemistry of MNs derived from control and ALS patient iPSCs showed that MNs produced by this method are useful for research on MNDs.

We also showed via time-lapse imaging that HB9-EGFP-positive cells emerged within 2 days after the transduction of SeV-L-N-I and that these cells extended neurites on day 3. Some of the cells gradually disappeared, perhaps because the SeV vectors may have had some cytotoxic effects or because we could not change the medium during time-lapse imaging.

There are still some challenges to be resolved. First, the number of infecting vectors may vary between individual cells. Second, the SeV vectors should be easily removable from the transduced cells after differentiation. Removable SeV vectors are now being developed. In addition, the homogeneity of the MNs needs to be further improved. Although further studies are required to determine whether this method is applicable to other types of neurons, we expect it will provide a new approach for research on neurodegenerative diseases.

In conclusion, we established a simple and useful method for differentiating human iPSCs into MNs with a single SeV vector encoding multiple transcription factors. This method will help to facilitate stem cell-based research on MNDs.

## Materials and Methods

The generation and use of human iPSCs was approved by the Ethics Committees of the respective departments, including Kyoto University. The procedures for generation of mouse iPSCs were performed in accordance with the Kyoto University Animal Institutional Guidelines, and all experiments were approved by the Center for iPS Cell Research and Application (CiRA) Animal Experiment Committee.

### Derivation of Human Fibroblasts and Generation of iPSCs

Human fibroblasts were obtained with written consent. The iPSCs were generated according to a method previously described.^7^ After selecting iPSC colonies, iPSCs were cultured and passaged on an SNL feeder layer. The medium was primate embryonic stem cell medium (ReproCELL) with 4 ng/mL basic fibroblast growth factor (Wako Chemicals) and 50 mg/mL penicillin and streptomycin. The medium was changed every day and iPSCs were passaged about once a week.

### Derivation of Mouse Embryonic Fibroblasts and Generation of iPSCs

Mouse embryonic fibroblasts (MEFs) were obtained from ALS model mice carrying mutant SOD1 (G93A)^37^ or mutant TDP-43 (A315T)^38^ or from littermate controls. Four reprogramming factors (Oct3/4, Sox2, Klf4, and c-Myc) were introduced into the MEFs using retroviral vectors as reported previously.^39^, ^40^ Mouse iPSCs were cultured on SNL feeder cells.

### Genotyping

The human SOD1 gene was amplified from genomic DNA by PCR and directly sequenced using a 3500xL Genetic Analyzer (Applied Biosystems).

### Transduction Ratio by SeV Vectors into ESCs/iPSCs

To decide the transduction ratio by SeV vectors, SeV-EGFP (ID Pharma) was transduced into control iPSCs. The iPSCs were treated with collagenase type IV, trypsin, and knockout serum replacement (CTK) dissociation solution (ReproCELL) for 2 min, dissociated to single cells with Accumax (Innovative Cell Technologies), and transferred onto a 96-well plate coated with Matrigel (Becton Dickinson). Cells were fixed on day 2 and day 4. Images were captured by In Cell Analyzer 6000 (GE Healthcare).

### Differentiation of MNs from Human ESCs/iPSCs Using SeV Vectors

ESCs/iPSCs were treated with CTK dissociation solution for 2 min and feeder cells were removed with PBS. Then ESCs/iPSCs were dissociated to single cells with Accumax, and they were transferred onto Matrigel-coated plates with MN medium containing a 1:1 mixture of Neurobasal medium (Thermo Fisher Scientific) and DMEM/F12 (Thermo Fisher Scientific), supplemented with 0.5% N2 (Thermo Fisher Scientific), 1% B27 (Thermo Fisher Scientific), 1 μM retinoic acid (Sigma-Aldrich), 1 μM smoothened agonist (Enzo Life Sciences), 10 ng/mL brain-derived neurotrophic factor (BDNF; R&D Systems), 10 ng/mL glial cell-derived neurotrophic factor (GDNF; R&D Systems), 10 ng/mL neurotrophin-3 (NT-3; R&D Systems), and 10 μM Y-27632 (Nacalai Tesque). At the same time, the ESCs/iPSCs were infected with SeV-L-N-I; SeV-L-N-I-EGFP; or combinations of SeV-L, SeV-N, and SeV-I (ID Pharma) on day 0. MOIs were 5 or 10. The transduction of SeV vectors to human ESCs/iPSCs was conducted just once. The number of cells per well was 5.0 × 10^4^ in 96-well plates and 1.0 × 10^6^ in 12-well plates. The medium was changed to MN medium without Y-27632 on day 1 and then changed every 3 days.

For phenotype assays, cells were treated with Accumax plus 10 μM Y-27632 and transferred onto poly-L-lysine- and Matrigel-coated glass dishes on day 7. For immunocytochemistry and qPCR analyses, cells were assessed on day 14.

### Differentiation of MNs from Mouse iPSCs Using an SeV Vector

The iPSCs were trypsinized into single cells and plated on Matrigel-coated plates with MN medium. At the same time, the iPSCs were infected with SeV-L-N-I (ID Pharma) on day 0. The MOI was 5 because mouse iPSCs were damaged at an MOI of 10. The medium was changed to MN medium without Y-27632 on day 1 and day 4. Cells were assessed by immunocytochemistry on day 6.

### RNA Extraction, cDNA Synthesis, and qPCR

RNA was isolated using RNeasy Mini Kit (QIAGEN) according to the manufacturer’s instructions. The cDNA was synthesized using the ReverTra Ace-α Kit (Toyobo). The qPCR was performed with SYBR Premix Ex TaqII (Takara) by the StepOne Plus instrument (Applied Biosystems). Primer sequences are described in Table S6.

### Co-culture of Human MNs with Human Myogenic Cells

The Hu5/E18 cell line was purchased from RIKEN BioResource Center. Hu5/E18 cells were maintained and differentiated as previously reported.^44^ Cells were maintained in DMEM with high glucose (Nacalai Tesque) containing 20% fetal bovine serum (Gibco). Cells were differentiated into human myocytes in DMEM containing 5 μg/mL holo-transferrin bovine (Sigma-Aldrich), 10 μg/mL insulin (bovine, Sigma-Aldrich), 10 nM sodium selenite (Sigma-Aldrich), and 2% horse serum (Gibco) 7 days before SeV-L-N-I transduction into iPSCs. The iPSCs were transduced with SeV-L-N-I on day 0, dissociated with Accumax plus 10 μM Y-27632, and then transferred onto Hu5/E18-cultured plates on day 7. The medium was changed to MN medium. Cells were fixed with 4% paraformaldehyde (pH 7.4) for 30 min on day 14 and assessed by immunocytochemistry.

### Electrophysiological Recordings

Human iPSCs were transduced with SeV-L-N-I vector on day 0 and plated onto astrocytes on day 7. Electrophysiological recording and analysis were performed under microscopy in combination with differential interference contrast (DIC) imaging on day 21, as previously described.^7^ During the electrophysiological recording, cells were maintained at 30°C and continuously superfused with oxygenated Krebs-Ringer solution consisting of 125 mM NaCl, 2.5 mM KCl, 2 mM CaCl_2_, 1 mM MgCl_2_, 26 mM NaHCO_3_, 1.25 mM NaH_2_PO_4_, and 20 mM glucose. To examine whether iPSC-derived MNs were functionally active, action potentials were measured in current-clamp mode with a potassium chloride-based electrode solution composed of 140 mM KCl, 2 mM MgCl_2_, 10 mM HEPES, and 1 mM EGTA, adjusted to pH 7.4 with NaOH. For the recording, an EPC 9 amplifier (HEKA) was used, and the data were analyzed with Patchmaster software (HEKA). Primary astrocytes were cultured from post-natal day (P)1 mouse in DMEM containing 10% FBS.

### Immunocytochemistry

Cells were fixed with 4% paraformaldehyde (pH 7.4) for 30 min. The cells were then permeabilized with 0.2% Triton X-100, and non-specific binding sites were blocked with Block Ace (Yukijirushi). Cells were incubated with primary antibodies at 4°C overnight and with secondary antibodies at room temperature for 1 hr. Fluorescent images were captured using IN Cell Analyzer 6000, and the percentage of MNs or neurons was calculated using IN Cell Developer Toolbox v1.9 (GE Healthcare). For phenotype assays, images were acquired by Delta Vision (GE Healthcare). The primary antibodies were as follows: HB9 (Developmental Studies Hybridoma Bank [DSHB], 1:200), Tuj1 (Covance, 1:2,000), Tuj1 (Chemicon, 1:500) for Figures 6A and 6C, ChAT (Chemicon, 1:100), HOXB4 (DSHB, 1:50), HOXC6 (Abcam, 1:200), HOXC9 (Abcam, 1:200), HOXC10 (Abcam, 1:2,000), misfolded SOD1 (MEDIMABS, B8H10, 1:200), misfolded SOD1 (MEDIMABS, A5C3, 1:200), TDP-43 (Proteintech, 1:200), human Nanog (ReproCELL, 1:500), SSEA4 (Millipore, 1:200), SSEA1 (Chemicon, 1:1,000), Nestin (Millipore, 1:200), GFAP (Dako, 1:2,000), Iba1 (Wako Pure Chemicals Industries, 1:500), CNPase (Cell Signaling Technology, 1:100), SOX17 (R&D Systems, 1:200), and αSMA (Dako, 1:500). Tuj1 (Chemicon) was used to co-immunostain with TDP-43.

### Time-Lapse Imaging

For time-lapse imaging, 35-mm glass-bottom dishes (MatTek) were coated with poly-L-lysine (Sigma-Aldrich) and Matrigel. Human iPSCs transduced by SeV-L-N-I were plated on the dishes on day 0. The medium was changed to FluoroBrite DMEM (Thermo Fisher Scientific) supplemented with 0.5% N2, 1% B27, 1 μM retinoic acid, 1 μM smoothened agonist, 10 ng/mL BDNF, 10 ng/mL GDNF, and 10 ng/mL NT-3 on day 1. Time-lapse imaging was started 24 hr after plating using BioStation IM-Q (Nikon). Images were captured every 30 min.

### Statistical Analysis

All data are shown as mean ± SEM. Data were analyzed by Student’s t test or one-way ANOVA followed by Dunnett’s post-hoc test; p values < 0.05 were considered significant. Statistical analyses were performed with SPSS version 21 (IBM).

## Author Contributions

H.I. conceived the study. K.G., K.I., and K.K. designed, conducted, and analyzed the experiments and prepared the figures. H.I. and M.I. discussed the vector design. K.G., K.I., K.K., and H.I. wrote the manuscript. K.M., K.A., N.N., M.I., and A.K. provided the materials. H.Y. and R.T. provided advice regarding the data and the manuscript. All authors reviewed the manuscript.

## Conflicts of Interest

M.I. is a board member of ID Pharma Co., Ltd.

## Supplemental Information

Document S1. Figures S1–S6 and Tables S1–S6

Movie S1. Time-Lapse Imaging of EGFP-Positive Cells Induced with a Single SeV Vector Encoding Lhx3, Ngn2, and Isl1 to HB9-EGFP Knockin Human iPSCs Time-lapse imaging was conducted from day 1. The images were captured every 30 min. Recording duration was 50 hr.

Document S2. Article plus Supplemental Information
