# Supplementary material for: Simple Derivation of Spinal Motor Neurons from ESCs/iPSCs Using Sendai Virus Vectors
Source: Mol Ther Methods Clin Dev. 2017 Jan 10;4:115–25. doi: 10.1016/j.omtm.2016.12.007 (PMC5363292; doi:10.1016/j.omtm.2016.12.007)
Supplement: Document S1. Figures S1–S6 and Tables S1–S6 [file mmc1.pdf]

OMTM, Volume *4*

Supplemental Information

Simple Derivation of Spinal Motor Neurons from ESCs/iPSCs Using Sendai Virus Vectors

Kazuya Goto, Keiko Imamura, Kenichi Komatsu, Kohnosuke Mitani, Kazuhiro Aiba, Norio Nakatsuji, Makoto Inoue, Akihiro Kawata, Hirofumi Yamashita, Ryosuke Takahashi, and Haruhisa Inoue

Supplemental Information

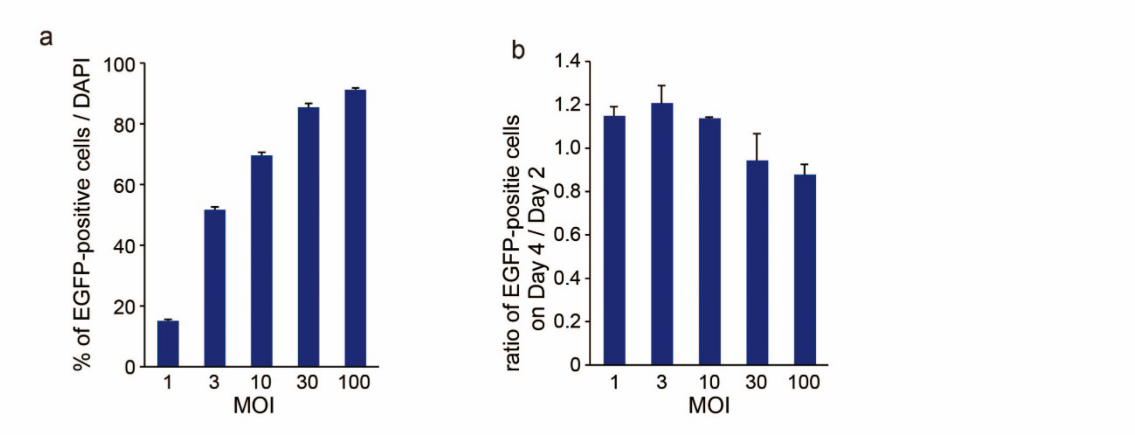

Fig. S1. Transduction of SeV-EGFP vector into iPSCs.

(a) Transduction efficiency of SeV-EGFP into iPSCs. Percentages of EGFP-positive cells on Day 2 were 15.1 ± 0.5%, 51.7 ± 1.0%, 69.6 ± 1.1%, 85.4 ± 1.3%, and 91.2 ± 0.6% at MOIs of 1, 3, 10, 30, and 100, respectively. Error bars are SEM, n = 3. (b) Ratios of EGFP-positive cells on Day 4 to Day 2 were 1.15 ± 0.04, 1.21 ± 0.08, 1.14 ± 0.01, 0.94 ± 0.12, and 0.88 ± 0.05 at MOIs of 1, 3, 10, 30, and 100, respectively. Error bars are SEM, n = 3.

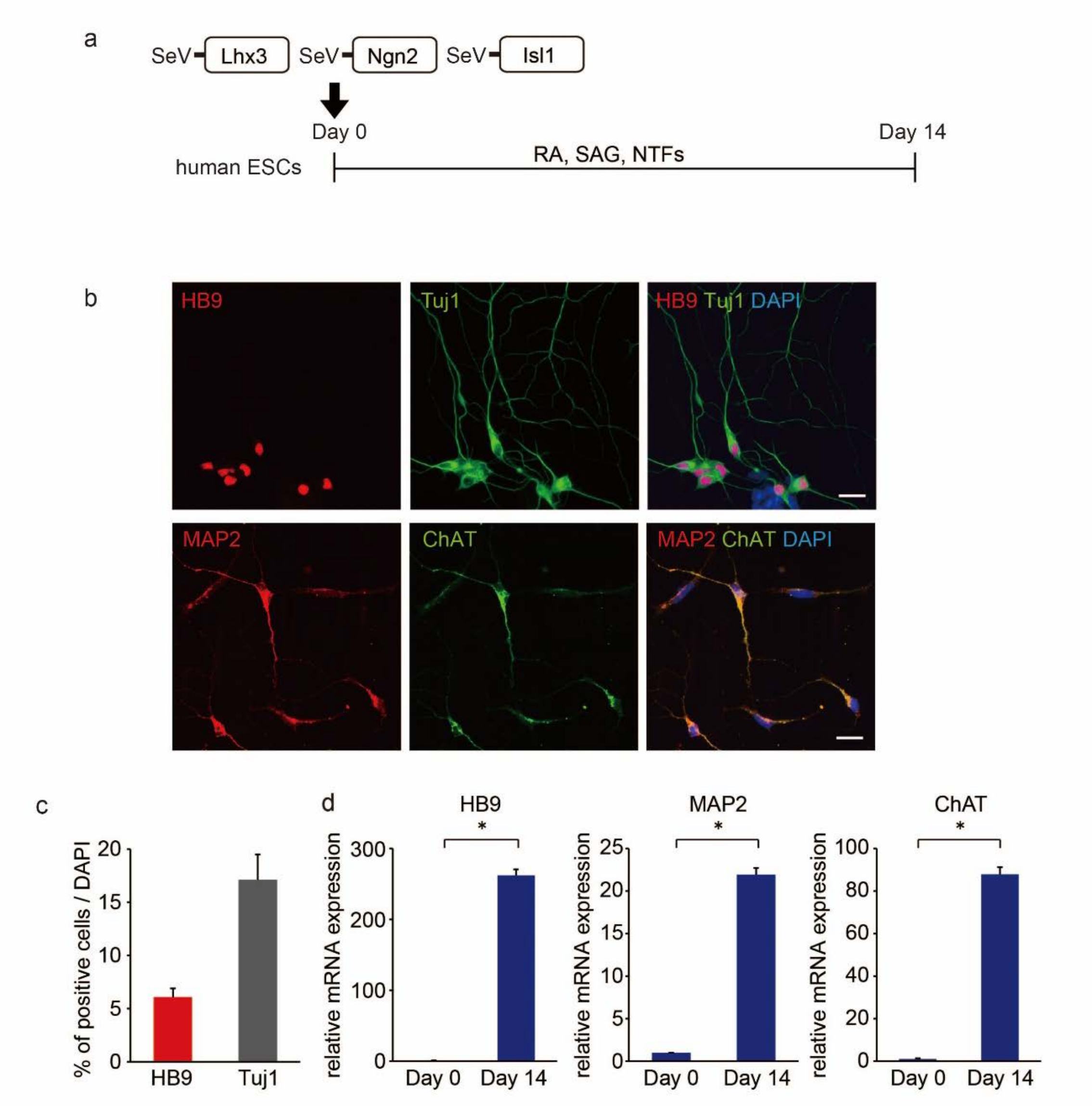

Figure S2. Differentiation of MNs from ESCs with three separate Sendai virus vectors.

(a) Outline of the experimental protocol to generate MNs using the single vectors SeV-L, SeV-N, and SeV-I in H9 ESCs. (b) Immunofluorescence staining showed the expression of MN markers (HB9 and ChAT) and neuronal markers (Tuj1 and MAP2). Scale bars, 20 μm. (c) Differentiation efficiency of MNs in SeV-infected cells. The percentages of HB9-positive and Tuj1-positive cells per total cells on Day 14 were 6.1 ± 0.8% and 17.1 ± 2.4%, respectively. (d) The qPCR analysis

of differentiated cells on Days 0 and 14 for MN markers (HB9 and ChAT) and neuronal marker (MAP2). Student’s t-test was used for statistical comparison. *$p < 0.05$ versus Day 0. Error bars are SEM, n = 3.

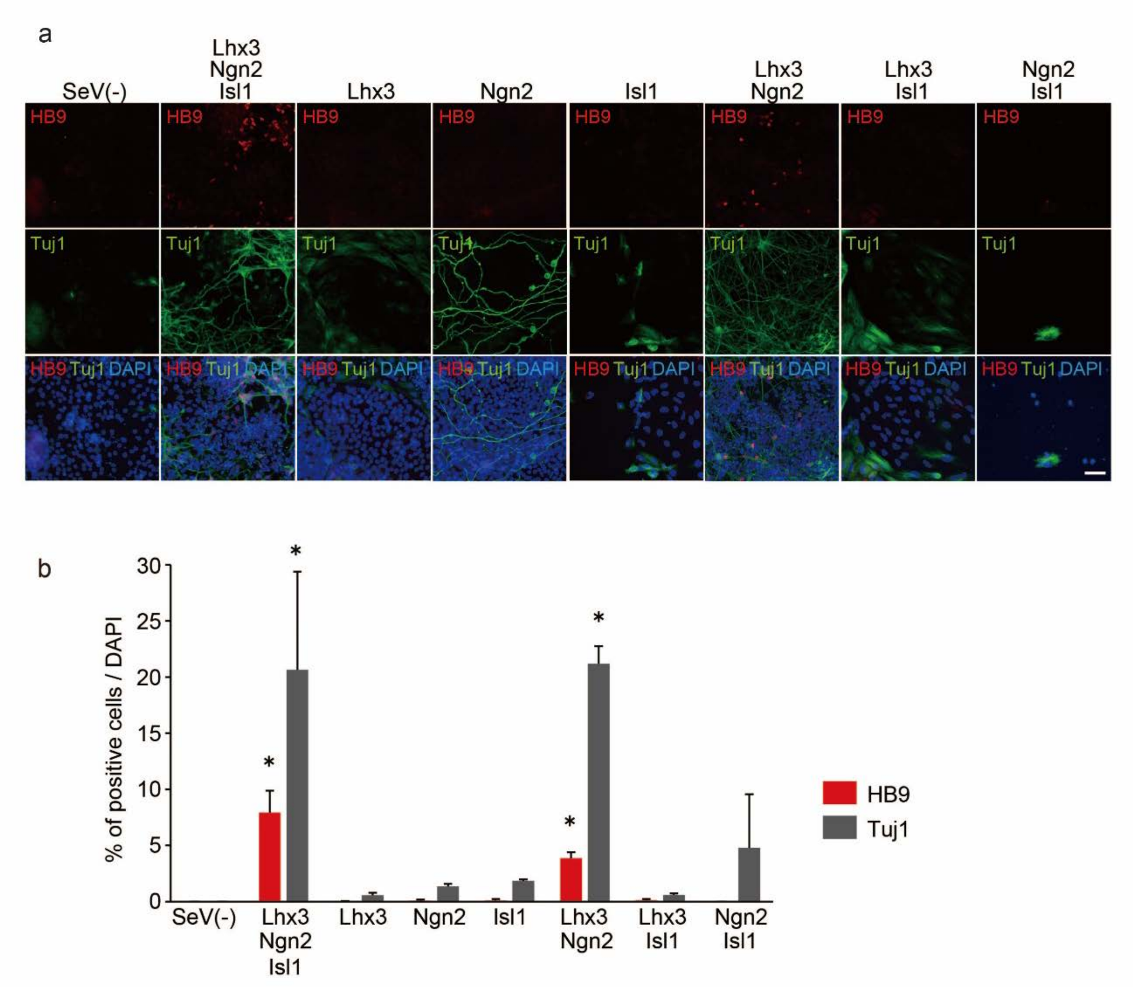

Figure S3. Comparison of combinations of three SeV vectors.

(a) Immunofluorescence staining of cells differentiated by various combinations of SeV-Lhx3 (Lhx3), SeV-Ngn2 (Ngn2), and SeV-Isl1 (Isl1) for HB9 (*top*) and Tuj1(*middle*); bottom row shows a merging of these images along with DAPI-stained nuclei (*blue*). Scale bar, 40 µm. (b) Percentages of HB9- (MNs; red) and Tuj1- (neurons; gray) positive cells derived by various combinations of the transcription factors. Data were analyzed by one-way ANOVA and Dunnett's post-hoc analysis. *$p < 0.05$. Error bars are SEM, n = 3.

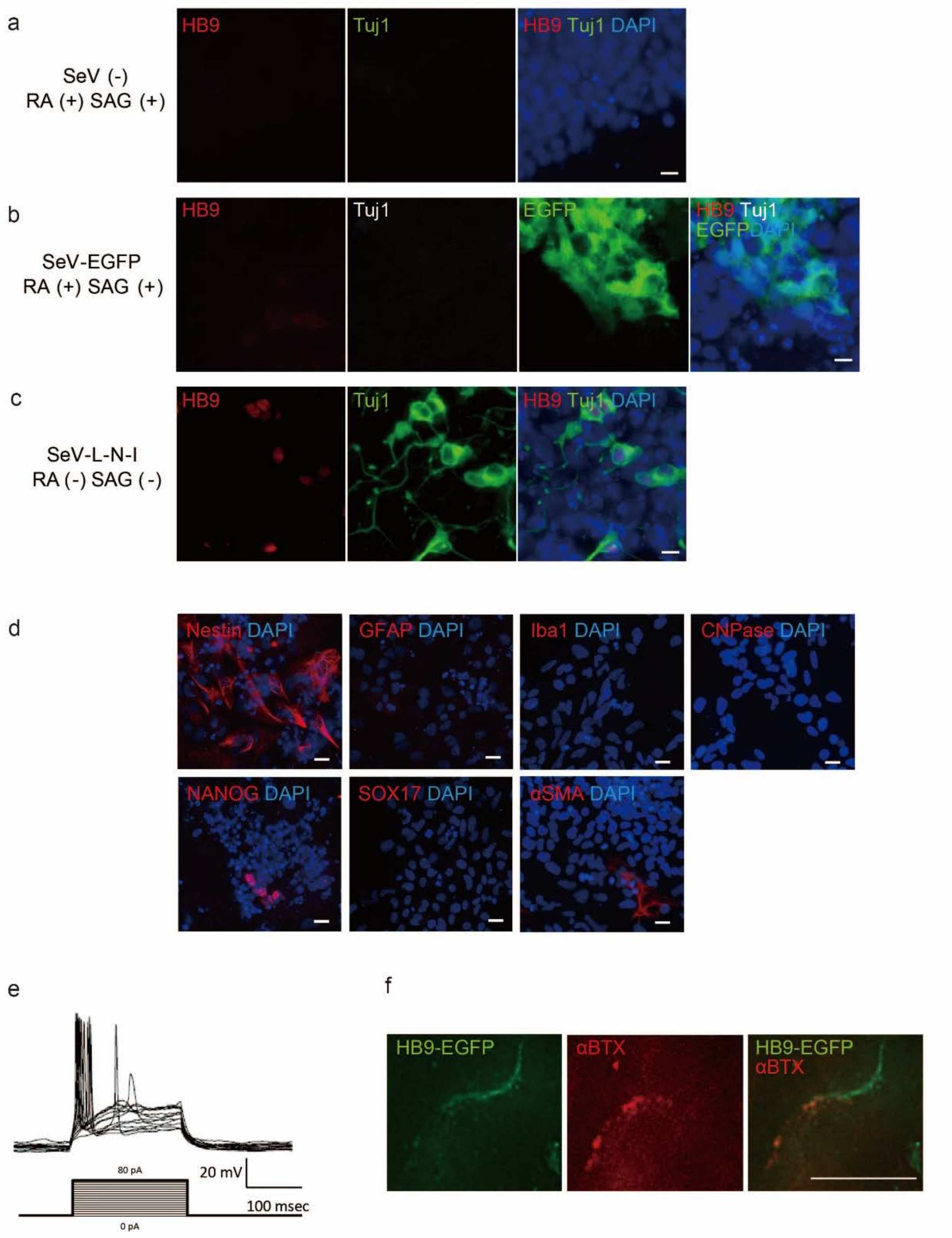

Fig. S4. Further analysis of cell culture by a single SeV vector encoding Lhx3, Ngn2, and Isl1.

(a) Differentiation of iPSCs using the current protocol without SeV vector. Tuj1- or HB9-positive cells were not observed. Scale bar, 20 μm. (b) Differentiation of iPSCs using the current protocol with SeV-EGFP vector. Tuj1- or HB9-positive cells were not observed. Scale bar, 20 μm. (c) Immunostaining of cells with SeV-L-N-I without RA and SAG. HB9-positive cells were 48.5 ± 1.5% of neurons. Scale bar, 20 μm. (d) Immunostaining for Nestin, GFAP, Iba1, CNPase, NANOG, SOX17, and αSMA on Day 14. The majority of non-neural cells were positive for Nestin. The number of Nestin-positive, NANOG-positive, and αSMA-positive cells in DAPI-stained total cells were 67.8 ± 2.7%, 0.49 ± 0.26%, and 0.27 ± 0.11%, respectively. Stainings for GFAP, SOX17, Iba1, and CNPase were negative. Scale bars, 20 μm. (e) Current-clamp recordings of action potentials on Day 21. (f) Double immunostaining against GFP for MNs and α-bungarotoxin for acetylcholine receptors on Day 21. Scale bar, 10 μm.

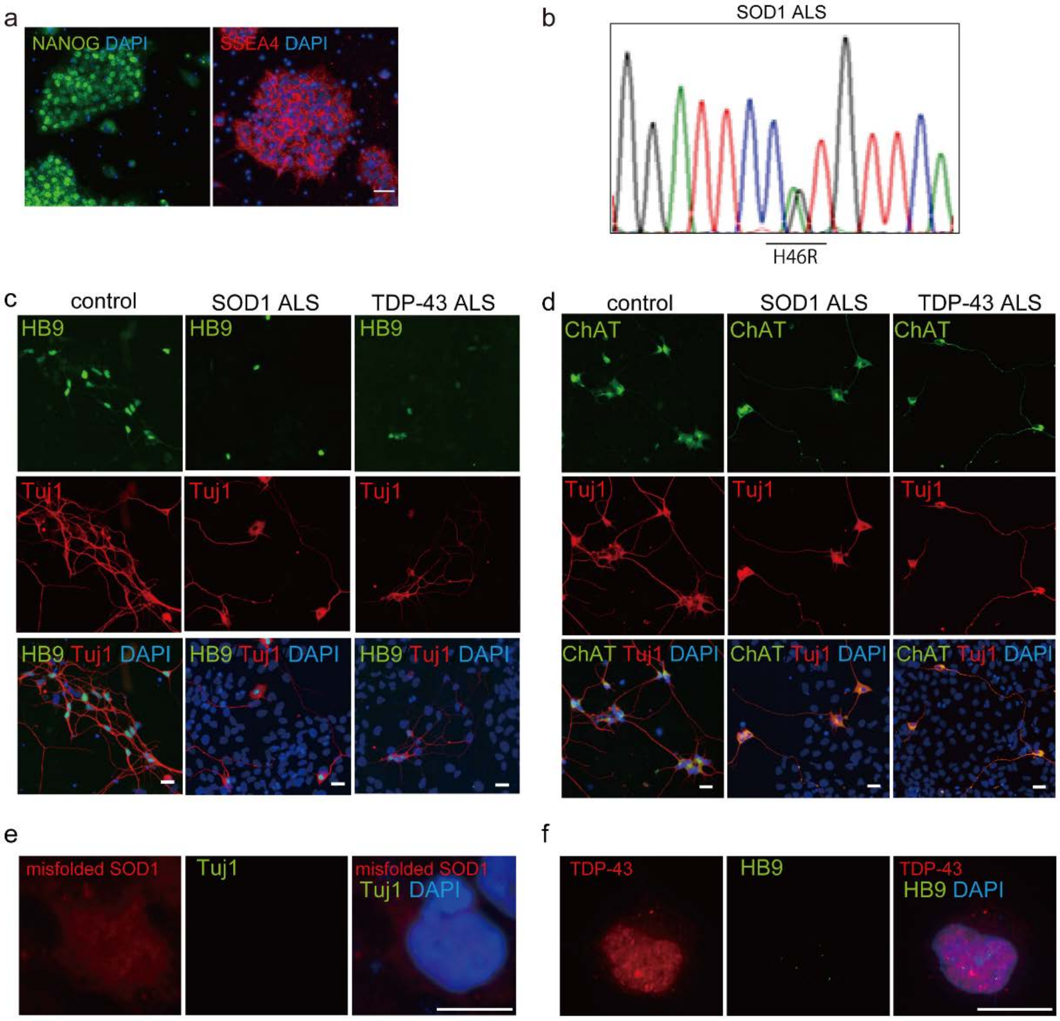

Figure S5. Differentiation of MNs from human control, SOD1-ALS and TDP-43-ALS iPSCs using a single SeV vector encoding Lhx3, Ngn2, and Isl1

(a) Immunofluorescence staining for NANOG and SSEA4 in human iPSCs derived from an SOD1-ALS patient. Scale bar, 30 µm. (b) Genomic DNA sequences for SOD1 mutation in human SOD1-ALS iPSCs. (c) Immunofluorescence staining of Tuj1 and HB9 in cells induced *via* SeV-L-N-I. Scale bars, 20 µm. (d) Immunofluorescence staining of Tuj1 and ChAT in cells induced *via* SeV-L-N-I. Scale bars, 20 µm. (e) SOD1-ALS iPSC-derived non-MNs were immunostained

with misfolded SOD1 and Tuj1 antibodies. Scale bar, 10 µm. (f) TDP-43-ALS iPSC-derived non-MNs were immunostained with TDP-43 and HB9 antibodies. Scale bar, 10 µm.

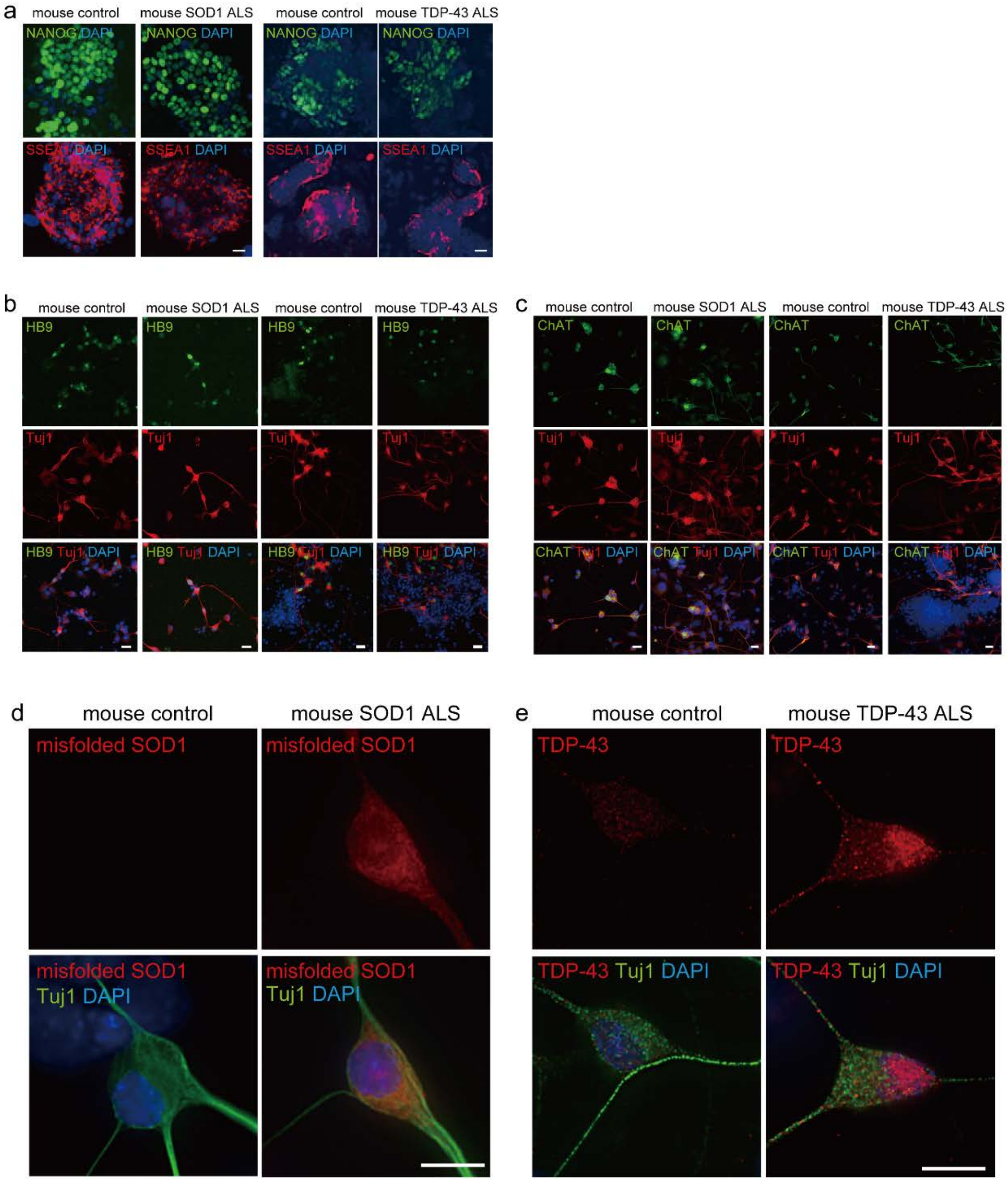

Figure S6. Phenotypes of SOD1-ALS and TDP-43-ALS mouse iPSC-derived neurons by a single SeV vector encoding Lhx3, Ngn2, and Isl1. (a) Immunofluorescence staining for NANOG and SSEA1 in control, SOD1-ALS and TDP-43-ALS mouse iPSCs. Scale bar, 30 µm. (b) Immunofluorescence staining of Tuj1 and HB9 in cells induced *via* SeV-L-N-I. Scale bars, 20 µm. (c) Immunofluorescence staining of Tuj1 and ChAT in cells induced *via* SeV-L-N-I.

Scale bar, 20 µm. (d) Control and SOD1-ALS mouse iPSC-derived MNs immunostained for misfolded SOD1 and Tuj1. Scale bar, 10 µm. (e) Control and TDP-43-ALS mouse iPSC-derived MNs were immunostained for TDP-43 and Tuj1. Scale bar, 10 µm.

Table S1. MN differentiation from iPSCs using signaling molecules.

Reference	Compounds for MN induction	Efficiency of MN differentiation	Days from iPSCs to MNs
Dimos et al., 2008	RA, SHH agonist	20% (HB9+ / all cells)	38 days
Ebert et al., 2009	RA, SHH	12.6 ± 2.2%, 9.5 ± 2.4% (ChAT+ and Tuj1+ / total cells)	28 days
Karumbayaram et al., 2009	RA, SHH	28.2 ± 5.7%, 33.6 ± 12% (ISL1+ / Tuj1+)	35-49 days
Zeng et al., 2010	RA, SHH	～20% (HB9+ / total cells)	35 days
Mitne-Neto et al., 2011	RA, SHH	～5% (HB9+ / total cells)	49 days
Amoroso et al., 2013	SB431542, LDN193189, RA, SAG, PUR	27 ± 1% (HB9+ or ISL1+ / DAPI)	21 days
Sareen et al., 2013	ATRA, purmorphamine	33-45% (SMI32+ / total cells)	49 days
Qu et al., 2014	Compound C, RA, SHH,	～2%, 51.6 ± 5.7% (HB9+ / DAPI)	18 days
Grunseich et al., 2014	SB431542, LDN193189, RA, SAG, PUR	20-30% (HB9+ / total cells)	21-30 days
Maury et al., 2015	SB431542, LDN193189, SAG, RA,CHIR99021, FGF2, DAPT	74% (HB9+ and ISL1+ / neurons)	14 days
Du et al., 2015	SB431542, DMH1, PUR, RA,CHIR99021, CpdE	91 ± 6% (ChAT+ / neurons)	28 days

Abbreviations: RA, retinoic acid; SHH, sonic hedgehog; SAG, smoothened agonist; PUR, purmorphamine; ATRA, all-trans retinoic acid; FGF2, fibroblast growth factor 2; DAPT, N-[N-(3,5-difluorophenacetyl)-l-alanyl]-S-phenylglycine t-butyl ester; Cpd E, Compound E.

Table S2. MN differentiation from ESCs/iPSCs or fibroblasts using transcription factors (TFs).

Reference	Initial source	TFs for MN induction	Efficiency of MN differentiation	HB9 or Phox2b detected days from ESCs/iPSCs	Medium
Hester et al., 2011	Human ESCs/iPSC	Ngn2, Isl1, Lhx3	55% (HB9+ / DAPI)	21 days	3 types
Son et al., 2011	HEF	Ascl1, Brn2, Myt1l, Lhx3,Hb9, Isl1, Ngn2, NeuroD1	~0.01% (Hb9+ / HEF)	30 days	2 types
Son et al., 2011	MEF	Ascl1, Brn2, Myt1l, Lhx3, Hb9, Isl1, Ngn2	5-10% (Hb9+ / MEF)	5 days	2 types
Mazzioni et al., 2013	Mouse ESC	Ngn2, Isl1, Lhx3	99.82 ± 0.17 (Hb9+ / transgenic cells)	1 day	2 types
Mazzioni et al., 2013	Mouse ESC	Ngn2, Isl1, Phox2a	99.03% (Phox2b+ / transgenic cells	3 days	2 types
This study	Human iPSC	Ngn2, Isl1, Lhx3	92.8 ± 1.2% (HB9+ / infected cells)	2 days	1 type

Abbreviations: ESC, embryonic stem cell; HEF, human embryonic fibroblast; MEF, mouse embryonic fibroblast.

Table S3. Comparison of virus vectors

Vector	Genetic material	Vector genome forms	Tropism	Packaging capacity
Retrovirus	RNA	Integrated	Dividing cells	~8kb
Lentivirus	RNA	Integrated	Broad	~8kb
Adenovirus	DNA	Episomal	Broad	~8-30kb
AAV	DNA	Episomal (90%)<br>Integrated (10%)	Broad	~5kb
Sendai virus	RNA	Episomal	Broad	~5kb

Abbreviations: AAV, adeno-associated virus

Table S4. List of human iPSC clones

human iPSC clone				
Clone name in this study	HB9-EGFP knockin	control	SOD1 ALS	TDP-43 ALS
Clone name at establishment	24-13	201B7	A30-1-1-1	A3411
Sex	Male	Female	Female	Female
Donor Age	New born	38	51	62
Origin	Fibroblast	Fibroblast	Fibroblast	Fibroblast
Reprogramming vector	Retrovirus	Retrovirus	Episomal	Episomal
Mutation	ー	ー	SOD1 (H46R)	TDP-43 (M337V)

Table S5. List of mouse iPSC clones

mouse iPSC clone				
Clone name in this study	control (SOD1)	mouse SOD1 ALS	control (TDP-43)	mouse TDP-43 ALS
Clone name at establishment	N-4	G-10	M7-5	M5-3
Origin	MEF	MEF	MEF	MEF
Reprogramming vector	Retrovirus	Retrovirus	Retrovirus	Retrovirus
Mutation	ー	SOD1 (G93A)	ー	TDP-43 (A315T)

Table S6. List of primers

Primers	Sequences (5' to 3')	Applications
HB9-Fw	tgcctaagatgcccgactt	qPCR
HB9-Rv	agctgctggctggtgaag	qPCR
ChAT-Fw	tgaaacctacctgatgagcaac	qPCR
ChAT-Rv	agcagaacatctccgtggtt	qPCR
MAP2-Fw	caggtggcggacgtgtgaaaattgagagtg	qPCR
MAP2-Rv	cacgctggatctgcctggggactgtg	qPCR
HOXB4-Fw	ctggatgcgcaaagttcac	qPCR
HOXB4-Rv	ttccttctccagctccaaga	qPCR
HOXC6-Fw	ccaggaccagaaagccagta	qPCR
HOXC6-Rv	ggtaccgcgagtagatctgg	qPCR
HOXC9-Fw	cagcaagcacaaagaggaga	qPCR
HOXC9-Rv	tccagcgtctggtacttgg	qPCR
HOXC10-Fw	gacacctcggataacgaagc	qPCR
HOXC10-Rv	cctcttcttccttccgctct	qPCR
GAPDH-Fw	tccactggcgtcttcacc	qPCR
GAPDH-Rv	ggcagagatgatgacccttt	qPCR

Movie S1. Time-lapse imaging of EGFP-positive cells induced with a single SeV vector encoding Lhx3, Ngn2, and Isl1 to HB9-EGFP knock-in human iPSCs.

Time-lapse imaging was conducted from Day 1. The images were captured every 30 minutes. Recording duration was 50 hours.
