# Supplementary figures and images for: Simple Derivation of Spinal Motor Neurons from ESCs/iPSCs Using Sendai Virus Vectors
Source: Mol Ther Methods Clin Dev. 2017 Jan 10;4:115–25. doi: 10.1016/j.omtm.2016.12.007 (PMC5363292; doi:10.1016/j.omtm.2016.12.007)

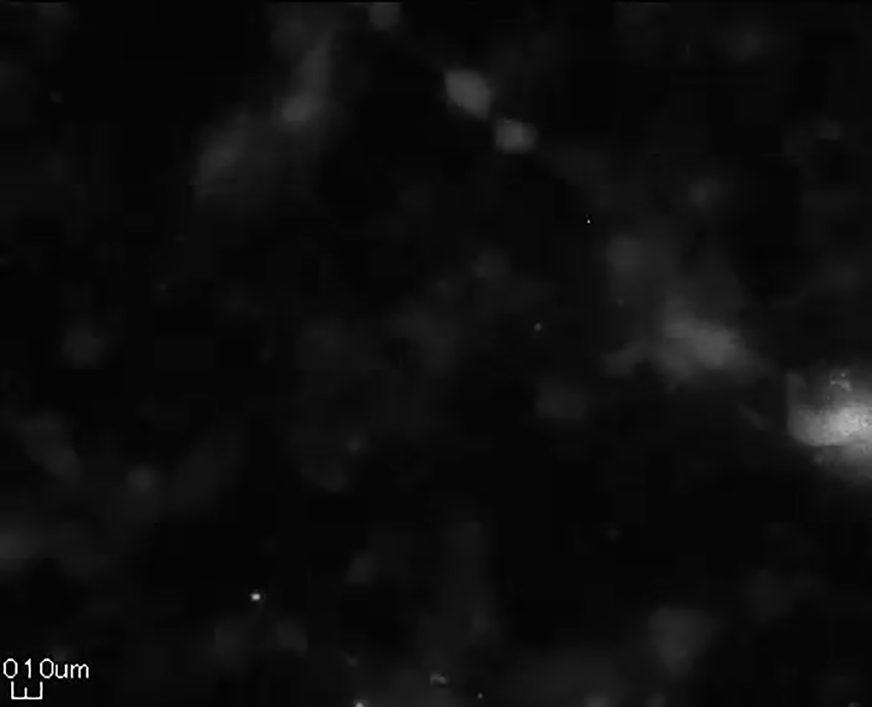

Supplement: Movie S1. Time-Lapse Imaging of EGFP-Positive Cells Induced with a Single SeV Vector Encoding Lhx3, Ngn2, and Isl1 to HB9-EGFP Knockin Human iPSCs — Time-lapse imaging was conducted from day 1. The images were captured every 30 min. Recording duration was 50 hr. [file mmc2.jpg]
