# Supplementary material for: Simple Derivation of Spinal Motor Neurons from ESCs/iPSCs Using Sendai Virus Vectors
Source: Mol Ther Methods Clin Dev. 2017 Jan 10;4:115–25. doi: 10.1016/j.omtm.2016.12.007 (PMC5363292; doi:10.1016/j.omtm.2016.12.007)
Supplement: Document S2. Article plus Supplemental Information [file mmc3.pdf]

Molecular Therapy
Methods & Clinical Development
Original Article

Simple Derivation of Spinal Motor Neurons from ESCs/iPSCs Using Sendai Virus Vectors

Kazuya Goto,[1,2,7] Keiko Imamura,[2,7] Kenichi Komatsu,[1,7] Kohnosuke Mitani,[3] Kazuhiro Aiba,[4] Norio Nakatsuji,[4] Makoto Inoue,[5] Akihiro Kawata,[6] Hirofumi Yamashita,[1] Ryosuke Takahashi,[1] and Haruhisa Inoue[2]

[1]Department of Neurology, Kyoto University Graduate School of Medicine, Kyoto 6068507, Japan; [2]Department of Cell Growth and Differentiation, Center for iPS Cell Research and Application (CiRA), Kyoto University, Kyoto 6068507, Japan; [3]Division of Gene Therapy and Genome Editing, Research Center for Genomic Medicine, Saitama Medical University, Saitama 3501241, Japan; [4]Institute for Integrated Cell-Material Sciences (WPI-iCeMS), Kyoto University, Kyoto 6068501, Japan; [5]DNAVEC Center, ID Pharma Co., Ltd., Tsukuba 3002611, Japan; [6]Department of Neurology, Tokyo Metropolitan Neurological Hospital, Tokyo 1830042, Japan

Amyotrophic lateral sclerosis (ALS) is a progressive and fatal degenerative disorder of motor neurons (MNs). Embryonic stem cells (ESCs)/induced pluripotent stem cells (iPSCs) now help us to understand the pathomechanisms of ALS via disease modeling. Various methods to differentiate ESCs/iPSCs into MNs by the addition of signaling molecules have been reported. However, classical methods require multiple steps, and newer simple methods using the transduction of transcription factors run the risk of genomic integration of the vector genes. Heterogeneity of the expression levels of the transcription factors also remains an issue. Here we describe a novel approach for differentiating human and mouse ESCs/iPSCs into MNs using a single Sendai virus vector encoding three transcription factors, LIM/homeobox protein 3, neurogenin 2, and islet-1, which are integration free. This single-vector method, generating HB9-positive cells on day 2 from human iPSCs, increases the ratio of MNs to neurons compared to the use of three separate Sendai virus vectors. In addition, the MNs derived via this method from iPSCs of ALS patients and model mice display disease phenotypes. This simple approach significantly reduces the efforts required to generate MNs, and it provides a useful tool for disease modeling.

INTRODUCTION

Amyotrophic lateral sclerosis (ALS), the most common and severe form of motor neuron diseases (MNDs), causes progressive muscle weakness and leads to death within several years. Vast amounts of findings concerning ALS have been reported, but the key mechanisms responsible for the disease are still not fully understood, hampering treatment. Consequently, the only FDA-approved drug, riluzole, was reported to prolong patient lifespan by just a few months.[1] The establishment of induced pluripotent stem cells (iPSCs) offers a new approach to the study of MNDs and the discovery of new drugs.[2,3] In 2008, the first ALS patient iPSC-derived motor neurons (MNs) were generated.[4] Since then, many ALS iPSC studies have been reported,[4–22] and this technology is leading to new findings and therapeutic candidates for ALS.

MNs can be obtained from iPSCs, using signaling molecules such as retinoic acid (RA) and Sonic hedgehog (Shh) (Table S1).[4,5,12,23–29] These methods rely on developmental principles and require changing the combinations of signaling molecules at multiple steps, which is why some methods require more than 4 weeks to produce MNs. In contrast, Hester et al. reported a rapid differentiation method using adenoviral vectors that encode the transcription factors neurogenin 2 (Ngn2), islet-1 (Isl1), and LIM/homeobox protein 3 (Lhx3).[30] These three transcription factors were transduced into neural progenitor cells, and MNs were obtained 11 days after the transduction. Son et al. reported that mouse and human fibroblasts were converted directly into MNs using seven and eight transcription factors, respectively, encoded by retrovirus vectors.[31] In 2013, Mazzoni et al. generated doxycyclin-inducible mouse embryonic stem cell lines to obtain MNs[32] (Table S2). Methods that rely on transcription factors are simple and rapid; but, when we use them for research on MNDs, we have to consider the possibility of genomic integration of the vector genes. Vector gene integration into host genomes contains the risk of influencing the behaviors of the transduced cells. Moreover, when we transduce several transcription factors separately, the transduction ratio of each transcription factor varies between the cells, and the heterogeneity of the cells may influence the experimental results. Therefore, we decided to focus on Sendai virus (SeV) vectors[33,34] (Table S3), which never integrate into host genomes with highly efficient transduction and expression levels of the transgene(s), and we designed a single SeV vector that encodes Lhx3, Ngn2, and Isl1 to produce more homogeneous MNs. Here we report that MNs can be induced from ESCs/iPSCs using a single SeV vector encoding a combination of transcription factors and that ALS iPSC-derived MNs exhibit disease phenotypes.

RESULTS

Differentiation of Human iPSCs into MNs with Three Separate SeV Vectors

First, we differentiated human iPSCs into MNs as described in Figure 1A. To detect MNs easily, we used HB9-EGFP knockin human

Received 30 September 2016; accepted 28 December 2016;
http://dx.doi.org/10.1016/j.omtm.2016.12.007.

[7]These authors contributed equally to this work.

Correspondence: Haruhisa Inoue, Center for iPS Cell Research and Application (CiRA), Kyoto University, 53 Kawahara-cho, Shogoin, Sakyo-ku, Kyoto 606-8507, Japan.
E-mail: haruhisa@cira.kyoto-u.ac.jp

This is an open access article under the CC BY license (http://creativecommons.org/licenses/by/4.0/).

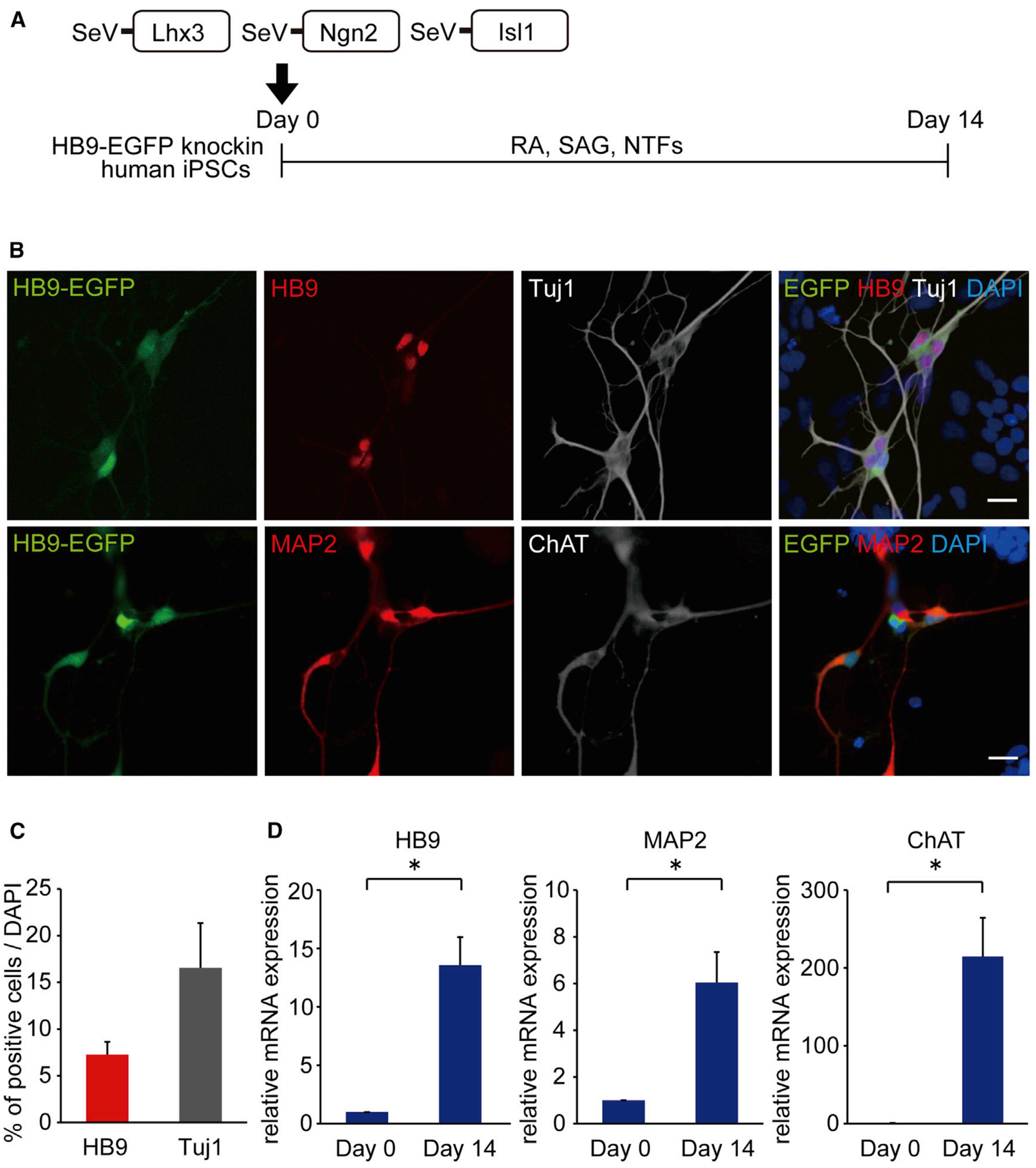

Figure 1. Differentiation of MNs with Three Separate Sendai Virus Vectors

(A) Outline shows the experimental procedure to generate MNs using SeV-Lhx3, SeV-Ngn2, and SeV-Isl1 from HB9-EGFP knockin human iPSCs. (B) Immunofluorescence staining of iPSC-derived MNs for the MN markers HB9 and ChAT and for the neuronal markers Tuj1 and MAP2 is shown. Scale bars, 20 μm. (C) The percentages of HB9-positive and Tuj1-positive cells per total cells were 7.3% ± 1.4% and 16.6% ± 4.8% on day 14, respectively. Error bars are SEM (n = 6). (D) The qPCR analysis of differentiated cells on days 0 and 14 for MN markers (HB9 and ChAT) and neuronal marker (MAP2) is shown. Student's t test was used for statistical comparison (*p < 0.05). Error bars are SEM (n = 3).

iPSCs.[35] On day 0, iPSCs were seeded on Matrigel-coated dishes and the medium was changed from ESC medium to neurobasal medium with N2 and B27 supplements. RA, smoothened agonist (SAG), and neurotrophic factors (NTFs) also were added from day 0. For the differentiation to MNs, three separate vectors, SeV18+Lhx3/TS7ΔF (SeV-L), SeV18+Ngn2/TS7ΔF (SeV-N), and SeV18+Isl1/TS7ΔF (SeV-I) were transduced into human iPSCs. To test the transduction efficiency of the SeV vectors, we transduced SeV18+EGFP/TS7ΔF (SeV-EGFP) into control iPSCs at the multiplicity of infections (MOIs) of 1, 3, 10, 30, and 100. We observed

dose-dependent increases of EGFP-positive cells on day 2. However, the ratios of EGFP-positive cells on day 4 compared to day 2 decreased at MOIs of 30–100 (Figure S1). Thus, we chose an MOI of less than 30. On day 14, we observed HB9-positive neurons and Tuj1-positive neurons, and 7.3% ± 1.4% and 16.6% ± 4.8% of total cells were positive for HB9 and Tuj1, respectively (Figures 1B and 1C). The qPCR analysis showed increased expression levels of HB9, ChAT, and MAP2 (Figure 1D). We also confirmed via immunocytochemistry and qPCR analysis that MNs can be generated from human ESCs with these SeV vectors (Figure S2).

To determine which combination of Lhx3, Ngn2, and Isl1 best produces MNs from iPSCs, we transduced one to three of SeV-L, SeV-N, and SeV-I into human iPSCs, and we evaluated Tuj1 and HB9 expressions by immunocytochemistry. The combination of all three factors and the combination of SeV-L and SeV-N produced both Tuj1- and HB9-positive neurons. The percentage of MNs per neurons was 43.9% ± 6.6% with all three factors and 18.2% ± 1.1% with Lhx3 and Ngn2 (Figure S3). We, therefore, decided to use all three factors for the differentiation of MNs.

Differentiation to MNs by Lhx3, Ngn2, and Isl1 in a Single SeV Vector and Time-Lapse Imaging

To increase the percentage of MNs per neurons, we designed a single SeV vector encoding Lhx3, Ngn2, and Isl1 (SeV-L-N-I). Each transgene was connected with the transcription termination (E), trinucleotide intergenic (I), and transcription restart sequence (S) of the Sendai virus (EIS sequence). We examined the differentiation of MNs using this vector (Figure 2A). On day 14, we observed both HB9-positive neurons and ChAT-positive neurons, and 6.2% ± 1.6% of the total cells were neurons and 5.3% ± 1.5% were MNs. The percentage of MNs per neurons was 85.6% ± 1.7% (Figures 2B and 2C). We confirmed no MNs were obtained without SeV-L-N-I or with SeV-EGFP vector only using the current protocol. Without RA and SAG, HB9-positive cells were 48.5% ± 1.5% of neurons (Figures S4A–S4C). We also analyzed populations other than neurons (Figure S4D). The qPCR analysis showed increased expression levels of HB9, ChAT, and MAP2 (Figure 2D). By electrophysiological patch-clamp analysis, we observed the action potentials of generated MNs when co-cultured with primary astrocytes (Figure S4E). When MNs were co-cultured with human myocytes differentiated from a human myogenic cell line, Hu5/E18, the formation of neuromuscular junctions was confirmed by co-localization of HB9-EGFP-positive neurites with α-bungarotoxin-stained acetylcholine receptors (Figure S4F).

To capture when HB9-positive cells emerge, we conducted time-lapse imaging analysis using HB9-EGFP knockin iPSCs. Time-lapse imaging of EGFP was started on day 1, and EGFP-positive cells were observed on day 2. The number of EGFP-positive cells gradually increased, but some of them disappeared as time passed. On day 3, neuron-like morphology was observed (Movie S1).

Differentiation to MNs by a Single SeV Vector Encoding Lhx3, Ngn2, Isl1, and EGFP

Since the efficiency for the differentiation of neural lineage appeared low based on the total number of cells, to investigate the MN and neuron differentiation efficiency in SeV-infected cells, we designed an SeV-L-N-I-EGFP vector, which could label SeV-infected cells, and we transduced the three factors into iPSCs (Figure 3A). We found that >90% of SeV-L-N-I-EGFP-infected cells had differentiated into MNs and neurons (Figures 3B and 3C). The qPCR analysis showed increased expression levels of HB9, ChAT, and MAP2 (Figure 3D). Then, to evaluate the terminal subtypes of MNs along the rostrocaudal axis of the spinal cord, we examined the expression of HOX genes by qPCR and immunostaining.[36] We observed increased mRNA expression of HOXB4, HOXC6, and HOXC9 on day 14, but the expression change of HOXC10 was not significant. Immunostaining showed that HOXC6-positive cells were about 60% of EGFP-positive cells (Figure 4). To analyze the efficiency for the differentiation to MNs from neural lineage cells, we transduced SeV-L-N-I-EGFP after treatment with dorsomorphin and SB431542 for 4 or 7 days (Figure 5). These results showed that the differentiation to MNs from neural lineage cells increased the number of HB9-positive cells compared to that from iPSCs.

SOD1-ALS and TDP-43-ALS MNs, Differentiated by a Single SeV Vector Encoding Lhx3, Ngn2, and Isl1, Exhibit Disease-Specific Phenotypes

To confirm that our method is applicable to the research of MNDs, we generated human iPSCs from the fibroblasts of a familial ALS patient with mutant superoxide dismutase 1 (SOD1 ALS) by transducing the four transcription factors Oct3/4, Sox2, Klf4, and c-Myc, as previously reported[3,7] (Table S4). The iPSCs were examined immunocytochemically for the ESC markers SSEA4 and NANOG (Figure S5A), and they were confirmed to retain the SOD1 gene mutation (Figure S5B). The generation of another familial ALS patients (mutant TAR DNA-binding protein, 43 kDa [TDP-43]-mediated ALS [TDP-43 ALS]), as well as control-derived iPSC lines, was reported previously.[7] When we differentiated human ALS iPSCs into MNs using SeV-L-N-I (Figures S5C and S5D), SOD1-ALS iPSC-derived neurons presented an accumulation of misfolded SOD1 (Figures 6A and 6B), and TDP-43-ALS iPSC-derived neurons exhibited cytosolic TDP-43 aggregation (Figures 6C and 6D). These cellular phenotypes were not specific to MNs (Figures S5E and S5F).

Next, we generated iPSCs from embryonic fibroblasts of ALS model mice carrying mutant SOD1[37] or mutant TDP-43[38] or from littermate controls by transducing Oct3/4, Sox2, Klf4, and c-Myc, as previously reported[39,40] (Figure S6A; Table S5). We differentiated the iPSCs into MNs to examine their phenotypes via immunocytochemistry (Figures S6B and S6C). MNs derived from mouse SOD1-ALS iPSCs were positive for misfolded SOD1, while those derived from mouse control iPSCs were negative (Figure S6D). MNs derived from mouse TDP-43-ALS iPSCs did not display the cytosolic aggregates of TDP-43

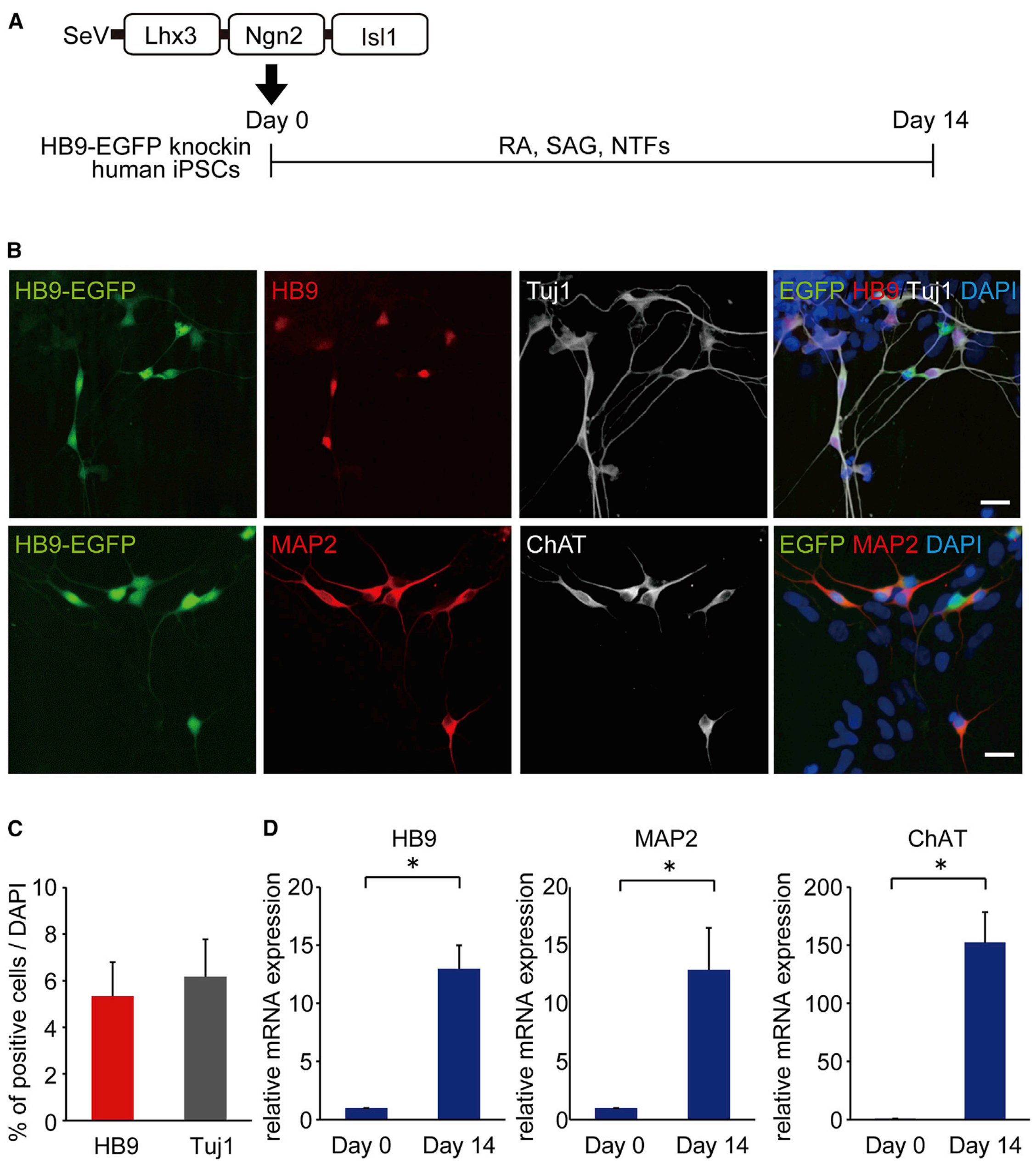

Figure 2. Differentiation of MNs with a Single SeV Vector Encoding Lhx3, Ngn2, and Isl1

(A) Outline shows the experimental procedure to generate MNs from HB9-EGFP knockin human iPSCs using a single vector, SeV-Lhx3-Ngn2-Isl1 (SeV-L-N-I). (B) Immunofluorescence staining of differentiated cells for HB9, Tuj1, MAP2, and ChAT is shown. Scale bars, 20 μm. (C) The percentages of HB9-positive and Tuj1-positive cells per total cells on day 14 were 5.3% ± 1.5% and 6.2% ± 1.6%, respectively. Error bars are SEM (n = 3). (D) The qPCR analysis of the differentiated cells on days 0 and 14 for HB9, ChAT, and MAP2 is shown. Student's t test was used for statistical comparison (*p < 0.05). Error bars are SEM (n = 3).

(Figure S6E), which is consistent with a report on TDP-43-transgenic mice.[41]

DISCUSSION

Along with the development of stem cell technology, stem cell-derived MNs have been utilized for modeling MNDs in vitro. However, the heterogeneity of these MN populations presents a potential issue for disease modeling and analysis. To obtain more homogeneous MNs, we used a single SeV vector that encodes three transcription factors. SeV, known as murine parainfluenza virus type 1, is a negative sense, single-stranded RNA virus of the family *Paramyxoviridae*. SeV vectors are cytoplasmic RNA vectors that do not integrate

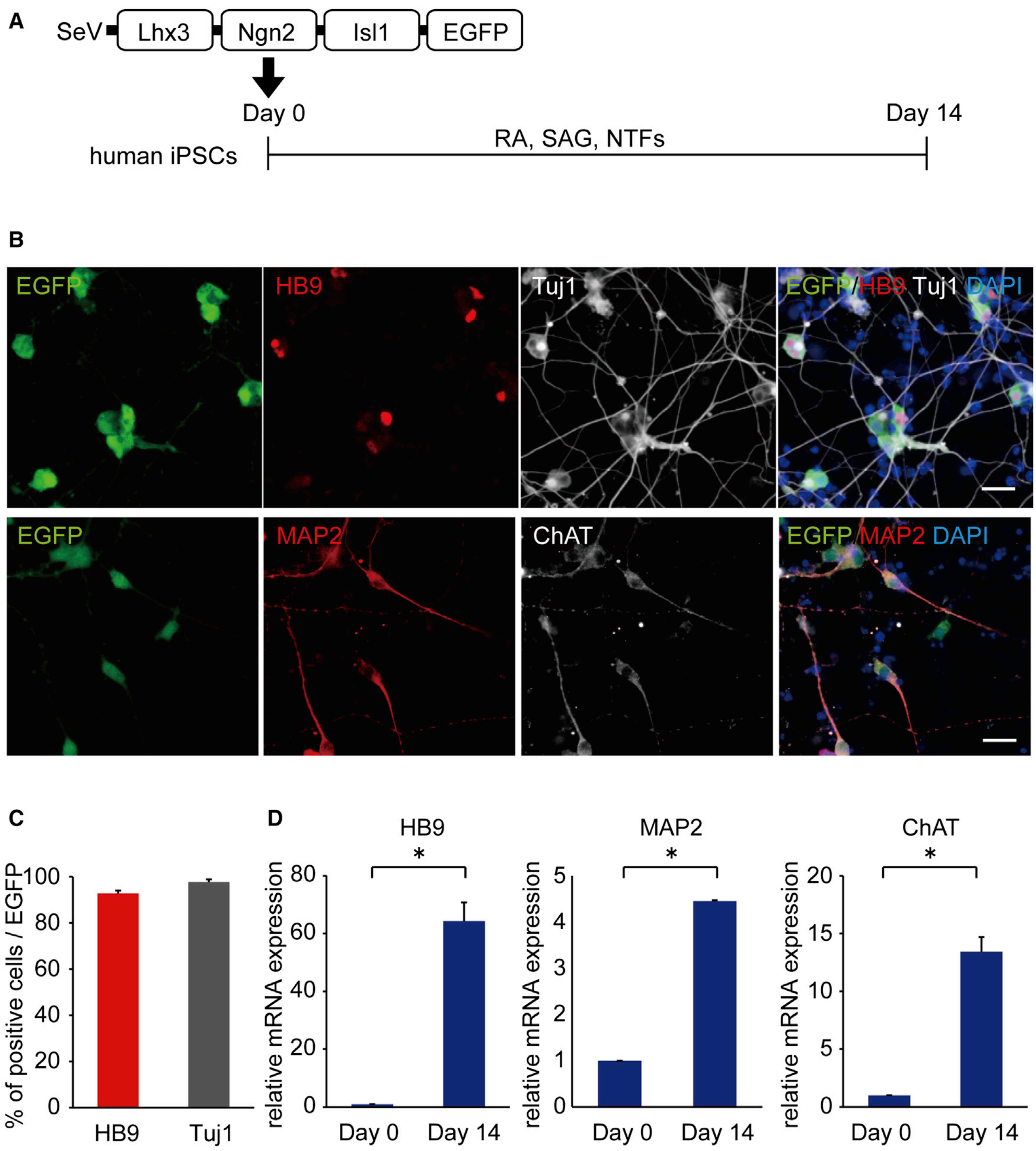

Figure 3. MN Differentiation Using a Single SeV Vector Encoding Lhx3, Ngn2, Isl1, and EGFP

(A) Outline shows the experimental procedure to generate MNs using SeV-L-N-I-EGFP from iPSCs. (B) Immunostaining for HB9, Tuj1, MAP2, and ChAT on day 14 is shown. Scale bars, 20 μm. (C) Differentiation efficiency of MNs in SeV-infected cells is shown. The percentages of HB9-positive and Tuj1-positive cells per EGFP-positive cells were 92.8% ± 1.2% and 97.7% ± 1.2% on day 14, respectively. Error bars are SEM (n = 3). (D) The qPCR analysis for HB9, MAP2, and ChAT is shown. Student's t test was used for statistical comparison (*p < 0.05). Error bars are SEM (n = 3).

into host genomes.[42] They can be transduced into both dividing and non-dividing cells, and short-term exposure is enough for efficient transduction.[43] SeV vectors can accommodate up to 5 kb of insertion.

The present study demonstrated that the ratio of MNs to neurons was higher when using a single SeV vector in comparison with three different SeV vectors for the transduction of Lhx3, Ngn2, and Isl1. The differentiation of neural lineage cells to MNs increases the percentage of HB9-positive cells per total cells compared to that of iPSCs. However, this method requires the dissociation and passage of cells and the change of compounds. On the other hand, the direct addition of a single vector to iPSCs is a very simple method, and the rapid differentiation of

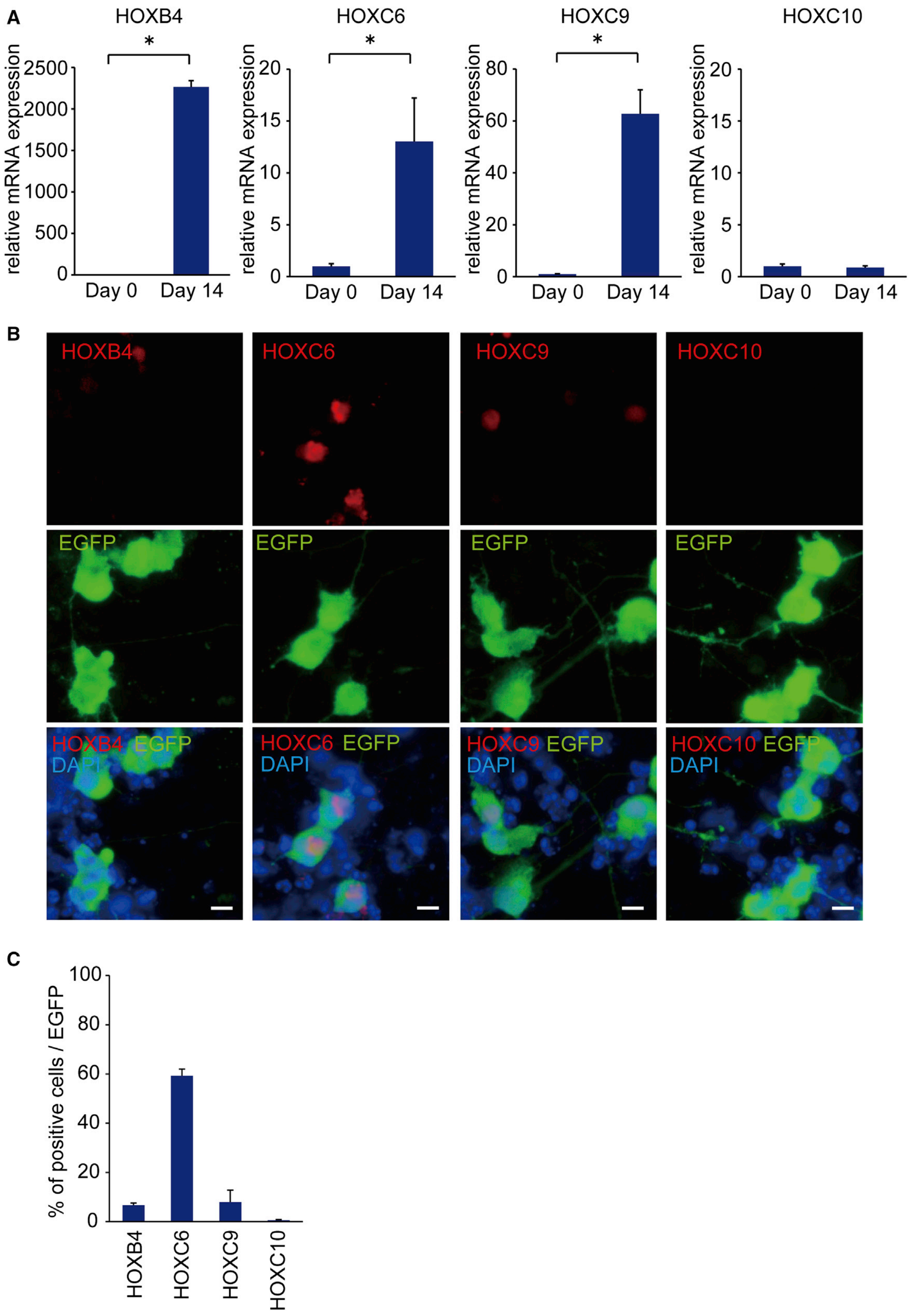

(legend on next page)

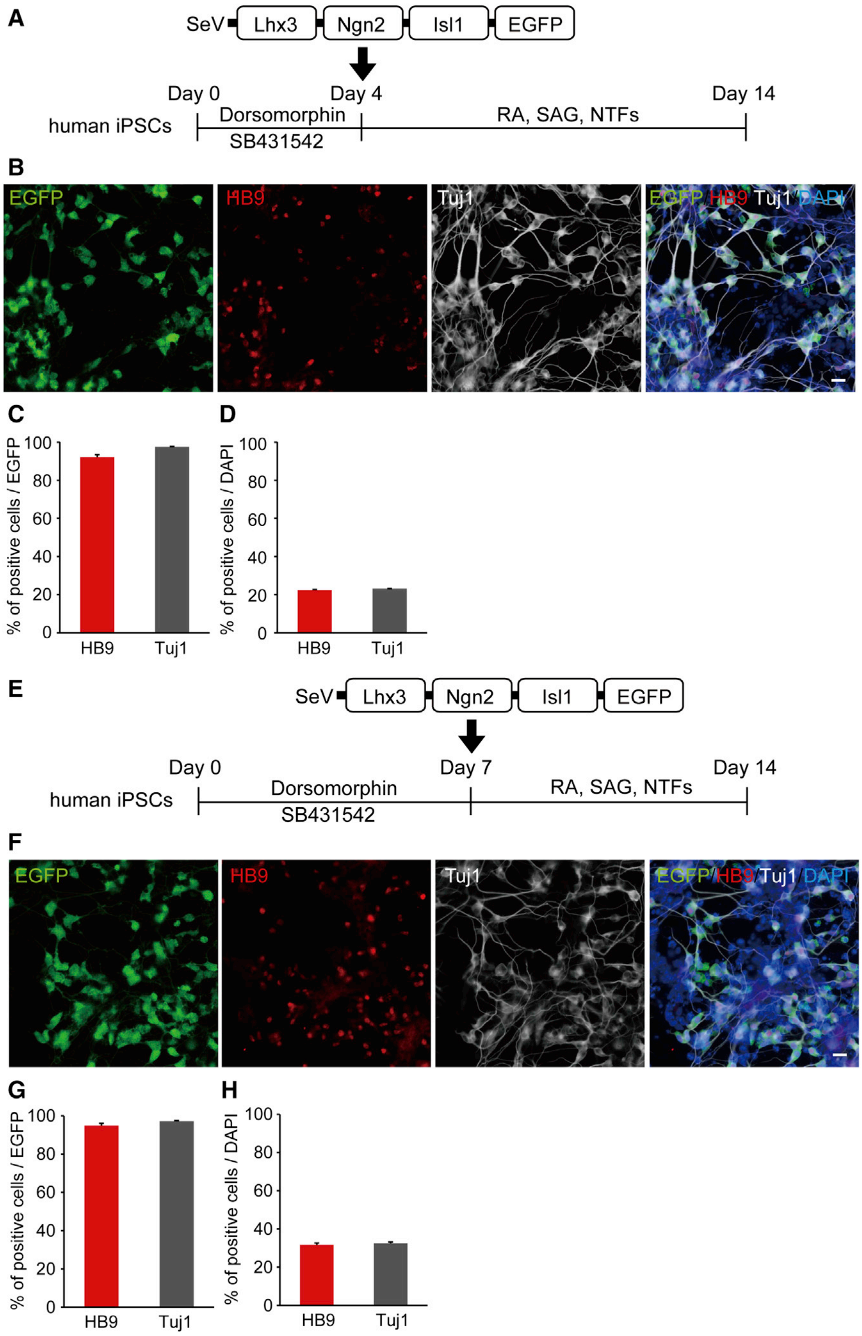

Figure 5. Differentiation of MNs from Neural Lineage Cells with a Single SeV Vector Encoding Lhx3, Ngn2, Isl1, and EGFP

(A) Outline shows the experimental procedure to generate MNs using SeV-L-N-I-EGFP from neural lineage cells treated with dorsomorhpin and SB431542 for 4 days. (B) Immunostaining for HB9 and Tuj1 on day 14 is shown. Scale bar, 20 μm. (C) Differentiation efficiency of MNs in SeV-infected cells is shown. The percentages of HB9-positive and Tuj1-positive cells per EGFP-positive cells were 94.0% ± 1.3% and 97.6% ± 0.2% on day 14, respectively. Error bars are SEM (n = 3). (D) The percentages of HB9-positive and Tuj1-positive cells per total cells were 22.2% ± 0.4% and 23.1% ± 0.1% on day 14, respectively. Error bars are SEM (n = 3). (E) Outline shows the experimental procedure to generate MNs using SeV-L-N-I-EGFP from neural lineage cells treated with dorsomorhpin and SB431542 for 7 days. (F) Immunostaining for HB9 and Tuj1 on day 14 is shown. Scale bar, 20 μm. (G) Differentiation efficiency to MNs of SeV-infected cells is shown. The percentages of HB9-positive and Tuj1-positive cells per EGFP-positive cells were 95.0% ± 1.1% and 97.3% ± 0.3% on day 14, respectively. Error bars are SEM (n = 3). (H) The percentages of HB9-positive and Tuj1-positive cells per total cells were 31.6% ± 1.0% and 32.4% ± 0.8% on day 14, respectively. Error bars are SEM (n = 3).

MNs is beneficial for research application. Moreover, immunocytochemistry of MNs derived from control and ALS patient iPSCs showed that MNs produced by this method are useful for research on MNDs.

We also showed via time-lapse imaging that HB9-EGFP-positive cells emerged within 2 days after the transduction of SeV-L-N-I and that these cells extended neurites on day 3. Some of the cells gradually disappeared, perhaps because the SeV vectors may have had some cytotoxic effects or because we could not change the medium during time-lapse imaging.

There are still some challenges to be resolved. First, the number of infecting vectors may vary between individual cells. Second, the SeV vectors should be easily removable from the transduced cells after differentiation. Removable SeV vectors are now being developed. In addition, the homogeneity of the MNs needs to be further improved. Although further studies are required to determine whether this method is applicable to other types of neurons, we expect it will provide a new approach for research on neurodegenerative diseases.

Figure 4. Characterization of MN Gene Expression Profile by a Single SeV Vector Encoding Lhx3, Ngn2, Isl1, and EGFP

(A) The qPCR analysis for HOXB4, HOXC6, HOXC9, and HOXC10 is shown. Student's t test was used for statistical comparison (*p < 0.05). Error bars are SEM (n = 3). (B) Immunostaining for HOXB4, HOXC6, HOXC9, and HOXC10 in MNs made by the SeV-L-N-I-EGFP vector is shown. Scale bars, 10 μm. (C) The percentage of HOX gene-positive cells per EGFP-positive cells is shown. Error bars are SEM (n = 3).

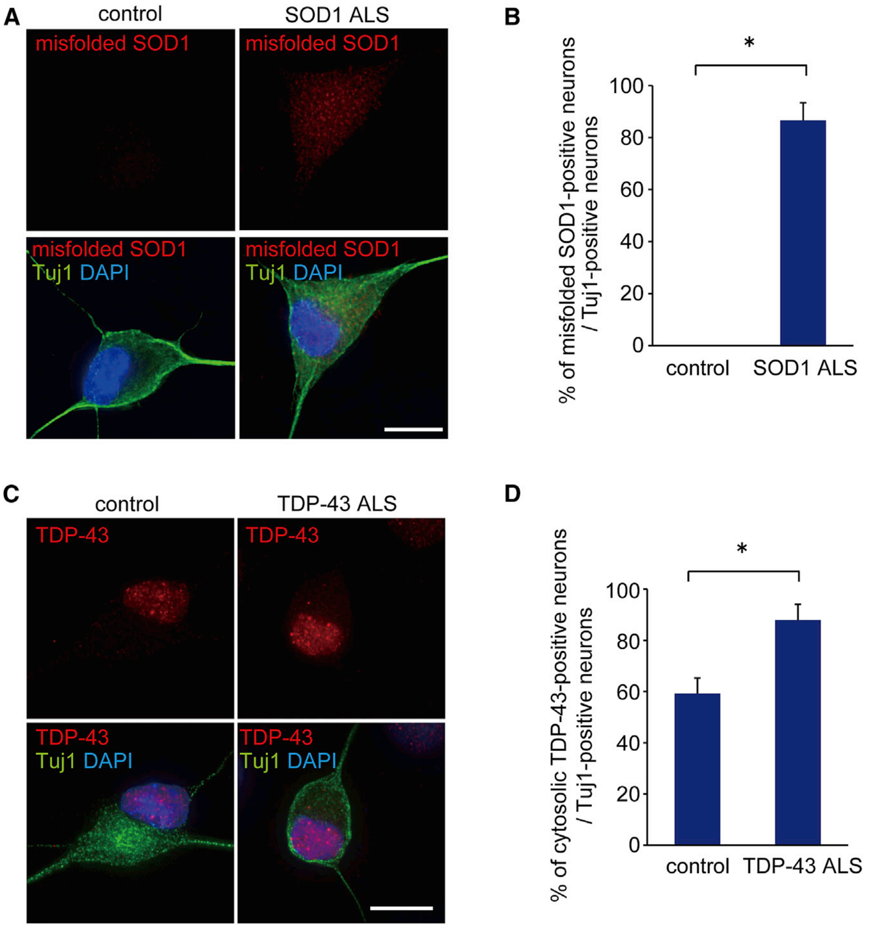

Figure 6. Phenotypes of SOD1-ALS and TDP-43-ALS iPSC-Derived Neurons by a Single SeV Vector Encoding Lhx3, Ngn2, and Isl1

(A) Immunostaining of misfolded SOD1 in control and SOD1-ALS iPSC-derived neurons is shown. Scale bar, 10 μm. (B) The percentages of misfolded SOD1-positive neurons in control and SOD1-ALS iPSC-derived neurons are shown. Student's t test was used for statistical comparison (*p < 0.05). Error bars are SEM (n = 3). (C) Immunostaining of TDP-43 in control and TDP-43-ALS iPSC-derived neurons is shown. Scale bar, 10 μm. (D) The percentages of TDP-43 aggregation-positive neurons in control and TDP-43-ALS iPSC-derived neurons are shown. Student's t test was used for statistical comparison (*p < 0.05). Error bars are SEM (n = 3).

In conclusion, we established a simple and useful method for differentiating human iPSCs into MNs with a single SeV vector encoding multiple transcription factors. This method will help to facilitate stem cell-based research on MNDs.

MATERIALS AND METHODS

The generation and use of human iPSCs was approved by the Ethics Committees of the respective departments, including Kyoto University. The procedures for generation of mouse iPSCs were performed in accordance with the Kyoto University Animal Institutional Guidelines, and all experiments were approved by the Center for iPS Cell Research and Application (CiRA) Animal Experiment Committee.

Derivation of Human Fibroblasts and Generation of iPSCs

Human fibroblasts were obtained with written consent. The iPSCs were generated according to a method previously described.[7] After selecting iPSC colonies, iPSCs were cultured and passaged on an SNL feeder layer. The medium was primate embryonic stem cell medium (ReproCELL) with 4 ng/mL basic fibroblast growth factor (Wako Chemicals) and 50 mg/mL penicillin and streptomycin. The medium was changed every day and iPSCs were passaged about once a week.

Derivation of Mouse Embryonic Fibroblasts and Generation of iPSCs

Mouse embryonic fibroblasts (MEFs) were obtained from ALS model mice carrying mutant SOD1 (G93A)[37] or mutant TDP-43 (A315T)[38] or from littermate controls. Four reprogramming factors (Oct3/4, Sox2, Klf4, and c-Myc) were introduced into the MEFs using retroviral vectors as reported previously.[39,40] Mouse iPSCs were cultured on SNL feeder cells.

Genotyping

The human SOD1 gene was amplified from genomic DNA by PCR and directly sequenced using a 3500xL Genetic Analyzer (Applied Biosystems).

Transduction Ratio by SeV Vectors into ESCs/iPSCs

To decide the transduction ratio by SeV vectors, SeV-EGFP (ID Pharma) was transduced into control iPSCs. The iPSCs were treated with collagenase type IV, trypsin, and knockout serum replacement (CTK) dissociation solution (ReproCELL) for 2 min, dissociated to single cells with Accumax (Innovative Cell Technologies), and transferred onto a 96-well plate coated with Matrigel (Becton Dickinson). Cells were fixed on day 2 and day 4. Images were captured by In Cell Analyzer 6000 (GE Healthcare).

Differentiation of MNs from Human ESCs/iPSCs Using SeV Vectors

ESCs/iPSCs were treated with CTK dissociation solution for 2 min and feeder cells were removed with PBS. Then ESCs/iPSCs were dissociated to single cells with Accumax, and they were transferred onto Matrigel-coated plates with MN medium containing a 1:1 mixture of Neurobasal medium (Thermo Fisher Scientific) and DMEM/F12 (Thermo Fisher Scientific), supplemented with 0.5% N2 (Thermo Fisher Scientific), 1% B27 (Thermo Fisher Scientific),

1 μM retinoic acid (Sigma-Aldrich), 1 μM smoothened agonist (Enzo Life Sciences), 10 ng/mL brain-derived neurotrophic factor (BDNF; R&D Systems), 10 ng/mL glial cell-derived neurotrophic factor (GDNF; R&D Systems), 10 ng/mL neurotrophin-3 (NT-3; R&D Systems), and 10 μM Y-27632 (Nacalai Tesque). At the same time, the ESCs/iPSCs were infected with SeV-L-N-I; SeV-L-N-I-EGFP; or combinations of SeV-L, SeV-N, and SeV-I (ID Pharma) on day 0. MOIs were 5 or 10. The transduction of SeV vectors to human ESCs/iPSCs was conducted just once. The number of cells per well was 5.0×10^4 in 96-well plates and 1.0×10^6 in 12-well plates. The medium was changed to MN medium without Y-27632 on day 1 and then changed every 3 days.

For phenotype assays, cells were treated with Accumax plus 10 μM Y-27632 and transferred onto poly-L-lysine- and Matrigel-coated glass dishes on day 7. For immunocytochemistry and qPCR analyses, cells were assessed on day 14.

Differentiation of MNs from Mouse iPSCs Using an SeV Vector

The iPSCs were trypsinized into single cells and plated on Matrigel-coated plates with MN medium. At the same time, the iPSCs were infected with SeV-L-N-I (ID Pharma) on day 0. The MOI was 5 because mouse iPSCs were damaged at an MOI of 10. The medium was changed to MN medium without Y-27632 on day 1 and day 4. Cells were assessed by immunocytochemistry on day 6.

RNA Extraction, cDNA Synthesis, and qPCR

RNA was isolated using RNeasy Mini Kit (QIAGEN) according to the manufacturer's instructions. The cDNA was synthesized using the ReverTra Ace-α Kit (Toyobo). The qPCR was performed with SYBR Premix Ex TaqII (Takara) by the StepOne Plus instrument (Applied Biosystems). Primer sequences are described in Table S6.

Co-culture of Human MNs with Human Myogenic Cells

The Hu5/E18 cell line was purchased from RIKEN BioResource Center. Hu5/E18 cells were maintained and differentiated as previously reported.[44] Cells were maintained in DMEM with high glucose (Nacalai Tesque) containing 20% fetal bovine serum (Gibco). Cells were differentiated into human myocytes in DMEM containing 5 μg/mL holo-transferrin bovine (Sigma-Aldrich), 10 μg/mL insulin (bovine, Sigma-Aldrich), 10 nM sodium selenite (Sigma-Aldrich), and 2% horse serum (Gibco) 7 days before SeV-L-N-I transduction into iPSCs. The iPSCs were transduced with SeV-L-N-I on day 0, dissociated with Accumax plus 10 μM Y-27632, and then transferred onto Hu5/E18-cultured plates on day 7. The medium was changed to MN medium. Cells were fixed with 4% paraformaldehyde (pH 7.4) for 30 min on day 14 and assessed by immunocytochemistry.

Electrophysiological Recordings

Human iPSCs were transduced with SeV-L-N-I vector on day 0 and plated onto astrocytes on day 7. Electrophysiological recording and analysis were performed under microscopy in combination with differential interference contrast (DIC) imaging on day 21, as previously described.[7] During the electrophysiological recording, cells were maintained at 30°C and continuously superfused with oxygenated Krebs-Ringer solution consisting of 125 mM NaCl, 2.5 mM KCl, 2 mM $CaCl_2$, 1 mM $MgCl_2$, 26 mM $NaHCO_3$, 1.25 mM NaH_2PO_4, and 20 mM glucose. To examine whether iPSC-derived MNs were functionally active, action potentials were measured in current-clamp mode with a potassium chloride-based electrode solution composed of 140 mM KCl, 2 mM $MgCl_2$, 10 mM HEPES, and 1 mM EGTA, adjusted to pH 7.4 with NaOH. For the recording, an EPC 9 amplifier (HEKA) was used, and the data were analyzed with Patchmaster software (HEKA). Primary astrocytes were cultured from post-natal day (P)1 mouse in DMEM containing 10% FBS.

Immunocytochemistry

Cells were fixed with 4% paraformaldehyde (pH 7.4) for 30 min. The cells were then permeabilized with 0.2% Triton X-100, and non-specific binding sites were blocked with Block Ace (Yukijirushi). Cells were incubated with primary antibodies at 4°C overnight and with secondary antibodies at room temperature for 1 hr. Fluorescent images were captured using IN Cell Analyzer 6000, and the percentage of MNs or neurons was calculated using IN Cell Developer Toolbox v1.9 (GE Healthcare). For phenotype assays, images were acquired by Delta Vision (GE Healthcare). The primary antibodies were as follows: HB9 (Developmental Studies Hybridoma Bank [DSHB], 1:200), Tuj1 (Covance, 1:2,000), Tuj1 (Chemicon, 1:500) for Figures 6A and 6C, ChAT (Chemicon, 1:100), HOXB4 (DSHB, 1:50), HOXC6 (Abcam, 1:200), HOXC9 (Abcam, 1:200), HOXC10 (Abcam, 1:2,000), misfolded SOD1 (MEDIMABS, B8H10, 1:200), misfolded SOD1 (MEDIMABS, A5C3, 1:200), TDP-43 (Proteintech, 1:200), human Nanog (ReproCELL, 1:500), SSEA4 (Millipore, 1:200), SSEA1 (Chemicon, 1:1,000), Nestin (Millipore, 1:200), GFAP (Dako, 1:2,000), Iba1 (Wako Pure Chemicals Industries, 1:500), CNPase (Cell Signaling Technology, 1:100), SOX17 (R&D Systems, 1:200), and αSMA (Dako, 1:500). Tuj1 (Chemicon) was used to co-immunostain with TDP-43.

Time-Lapse Imaging

For time-lapse imaging, 35-mm glass-bottom dishes (MatTek) were coated with poly-L-lysine (Sigma-Aldrich) and Matrigel. Human iPSCs transduced by SeV-L-N-I were plated on the dishes on day 0. The medium was changed to FluoroBrite DMEM (Thermo Fisher Scientific) supplemented with 0.5% N2, 1% B27, 1 μM retinoic acid, 1 μM smoothened agonist, 10 ng/mL BDNF, 10 ng/mL GDNF, and 10 ng/mL NT-3 on day 1. Time-lapse imaging was started 24 hr after plating using BioStation IM-Q (Nikon). Images were captured every 30 min.

Statistical Analysis

All data are shown as mean ± SEM. Data were analyzed by Student's t test or one-way ANOVA followed by Dunnett's post-hoc test; p values < 0.05 were considered significant. Statistical analyses were performed with SPSS version 21 (IBM).

SUPPLEMENTAL INFORMATION

Supplemental Information includes six figures, six tables, and one movie and can be found with this article online at http://dx.doi.org/10.1016/j.omtm.2016.12.007.

AUTHOR CONTRIBUTIONS

H.I. conceived the study. K.G., K.I., and K.K. designed, conducted, and analyzed the experiments and prepared the figures. H.I. and M.I. discussed the vector design. K.G., K.I., K.K., and H.I. wrote the manuscript. K.M., K.A., N.N., M.I., and A.K. provided the materials. H.Y. and R.T. provided advice regarding the data and the manuscript. All authors reviewed the manuscript.

CONFLICTS OF INTEREST

M.I. is a board member of ID Pharma Co., Ltd.

ACKNOWLEDGMENTS

We would like to express our sincere gratitude to all our coworkers and collaborators, including Noriko Endo, Mayumi Yamada, and Ruri Taniguchi for their valuable administrative support, and Takumi Kanaya, Takeo Yamamoto, Kaoru Takizawa, and Takashi Hironaka for their valuable technical support. We acknowledge Peter Karagiannis for providing critical reading. Funding for this project was received in part from the Program for Intractable Diseases Research utilizing disease-specific iPSCs from Japan Agency for Medical Research and Development (AMED) to H.I., from the Research Project for Practical Applications of Regenerative Medicine from AMED to H.I., from the grant for Core Center for iPS Cell Research of Research Center Network for Realization of Regenerative Medicine from AMED to H.I., and from the Daiichi Sankyo Foundation of Life Science to H.I.

REFERENCES

1. Bensimon, G., Lacomblez, L., and Meininger, V.; ALS/Riluzole Study Group (1994). A controlled trial of riluzole in amyotrophic lateral sclerosis. N. Engl. J. Med. *330*, 585–591.
2. Takahashi, K., and Yamanaka, S. (2006). Induction of pluripotent stem cells from mouse embryonic and adult fibroblast cultures by defined factors. Cell *126*, 663–676.
3. Takahashi, K., Tanabe, K., Ohnuki, M., Narita, M., Ichisaka, T., Tomoda, K., and Yamanaka, S. (2007). Induction of pluripotent stem cells from adult human fibroblasts by defined factors. Cell *131*, 861–872.
4. Dimos, J.T., Rodolfa, K.T., Niakan, K.K., Weisenthal, L.M., Mitsumoto, H., Chung, W., Croft, G.F., Saphier, G., Leibel, R., Goland, R., et al. (2008). Induced pluripotent stem cells generated from patients with ALS can be differentiated into motor neurons. Science *321*, 1218–1221.
5. Mitne-Neto, M., Machado-Costa, M., Marchetto, M.C., Bengtson, M.H., Joazeiro, C.A., Tsuda, H., Bellen, H.J., Silva, H.C., Oliveira, A.S., Lazar, M., et al. (2011). Downregulation of VAPB expression in motor neurons derived from induced pluripotent stem cells of ALS8 patients. Hum. Mol. Genet. *20*, 3642–3652.
6. Bilican, B., Serio, A., Barmada, S.J., Nishimura, A.L., Sullivan, G.J., Carrasco, M., Phatnani, H.P., Puddifoot, C.A., Story, D., Fletcher, J., et al. (2012). Mutant induced pluripotent stem cell lines recapitulate aspects of TDP-43 proteinopathies and reveal cell-specific vulnerability. Proc. Natl. Acad. Sci. USA *109*, 5803–5808.
7. Egawa, N., Kitaoka, S., Tsukita, K., Naitoh, M., Takahashi, K., Yamamoto, T., Adachi, F., Kondo, T., Okita, K., Asaka, I., et al. (2012). Drug screening for ALS using patient-specific induced pluripotent stem cells. Sci. Transl. Med. *4*, 145ra104.
8. Yao, X.L., Ye, C.H., Liu, Q., Wan, J.B., Zhen, J., Xiang, A.P., Li, W.Q., Wang, Y., Su, H., and Lu, X.L. (2013). Motoneuron differentiation of induced pluripotent stem cells from SOD1G93A mice. PLoS ONE *8*, e64720.
9. Yang, Y.M., Gupta, S.K., Kim, K.J., Powers, B.E., Cerqueira, A., Wainger, B.J., Ngo, H.D., Rosowski, K.A., Schein, P.A., Ackeifi, C.A., et al. (2013). A small molecule screen in stem-cell-derived motor neurons identifies a kinase inhibitor as a candidate therapeutic for ALS. Cell Stem Cell *12*, 713–726.
10. Serio, A., Bilican, B., Barmada, S.J., Ando, D.M., Zhao, C., Siller, R., Burr, K., Haghi, G., Story, D., Nishimura, A.L., et al. (2013). Astrocyte pathology and the absence of non-cell autonomy in an induced pluripotent stem cell model of TDP-43 proteinopathy. Proc. Natl. Acad. Sci. USA *110*, 4697–4702.
11. Almeida, S., Gascon, E., Tran, H., Chou, H.J., Gendron, T.F., Degroot, S., Tapper, A.R., Sellier, C., Charlet-Berguerand, N., Karydas, A., et al. (2013). Modeling key pathological features of frontotemporal dementia with C9ORF72 repeat expansion in iPSC-derived human neurons. Acta Neuropathol. *126*, 385–399.
12. Sareen, D., O'Rourke, J.G., Meera, P., Muhammad, A.K., Grant, S., Simpkinson, M., Bell, S., Carmona, S., Ornelas, L., Sahabian, A., et al. (2013). Targeting RNA foci in iPSC-derived motor neurons from ALS patients with a C9ORF72 repeat expansion. Sci. Transl. Med. *5*, 208ra149.
13. Donnelly, C.J., Zhang, P.W., Pham, J.T., Haeusler, A.R., Mistry, N.A., Vidensky, S., Daley, E.L., Poth, E.M., Hoover, B., Fines, D.M., et al. (2013). RNA toxicity from the ALS/FTD C9ORF72 expansion is mitigated by antisense intervention. Neuron *80*, 415–428.
14. Burkhardt, M.F., Martinez, F.J., Wright, S., Ramos, C., Volfson, D., Mason, M., Garnes, J., Dang, V., Lievers, J., Shoukat-Mumtaz, U., et al. (2013). A cellular model for sporadic ALS using patient-derived induced pluripotent stem cells. Mol. Cell. Neurosci. *56*, 355–364.
15. Wainger, B.J., Kiskinis, E., Mellin, C., Wiskow, O., Han, S.S., Sandoe, J., Perez, N.P., Williams, L.A., Lee, S., Boulting, G., et al. (2014). Intrinsic membrane hyperexcitability of amyotrophic lateral sclerosis patient-derived motor neurons. Cell Rep. *7*, 1–11.
16. Kiskinis, E., Sandoe, J., Williams, L.A., Boulting, G.L., Moccia, R., Wainger, B.J., Han, S., Peng, T., Thams, S., Mikkilineni, S., et al. (2014). Pathways disrupted in human ALS motor neurons identified through genetic correction of mutant SOD1. Cell Stem Cell *14*, 781–795.
17. Nizzardo, M., Simone, C., Rizzo, F., Ruggieri, M., Salani, S., Riboldi, G., Faravelli, I., Zanetta, C., Bresolin, N., Comi, G.P., and Corti, S. (2014). Minimally invasive transplantation of iPSC-derived ALDHhiSSCloVLA4+ neural stem cells effectively improves the phenotype of an amyotrophic lateral sclerosis model. Hum. Mol. Genet. *23*, 342–354.
18. Kondo, T., Funayama, M., Tsukita, K., Hotta, A., Yasuda, A., Nori, S., Kaneko, S., Nakamura, M., Takahashi, R., Okano, H., et al. (2014). Focal transplantation of human iPSC-derived glial-rich neural progenitors improves lifespan of ALS mice. Stem Cell Reports *3*, 242–249.
19. Chen, H., Qian, K., Du, Z., Cao, J., Petersen, A., Liu, H., Blackbourn, L.W., 4th, Huang, C.L., Errigo, A., Yin, Y., et al. (2014). Modeling ALS with iPSCs reveals that mutant SOD1 misregulates neurofilament balance in motor neurons. Cell Stem Cell *14*, 796–809.
20. Liu, X., Chen, J., Liu, W., Li, X., Chen, Q., Liu, T., Gao, S., and Deng, M. (2015). The fused in sarcoma protein forms cytoplasmic aggregates in motor neurons derived from integration-free induced pluripotent stem cells generated from a patient with familial amyotrophic lateral sclerosis carrying the FUS-P525L mutation. Neurogenetics *16*, 223–231.
21. Devlin, A.C., Burr, K., Borooah, S., Foster, J.D., Cleary, E.M., Geti, I., Vallier, L., Shaw, C.E., Chandran, S., and Miles, G.B. (2015). Human iPSC-derived motoneurons harbouring TARDBP or C9ORF72 ALS mutations are dysfunctional despite maintaining viability. Nat. Commun. *6*, 5999.
22. Lenzi, J., De Santis, R., de Turris, V., Morlando, M., Laneve, P., Calvo, A., Caliendo, V., Chiò, A., Rosa, A., and Bozzoni, I. (2015). ALS mutant FUS proteins are recruited into stress granules in induced pluripotent stem cell-derived motoneurons. Dis. Model. Mech. *8*, 755–766.
23. Ebert, A.D., Yu, J., Rose, F.F., Jr., Mattis, V.B., Lorson, C.L., Thomson, J.A., and Svendsen, C.N. (2009). Induced pluripotent stem cells from a spinal muscular atrophy patient. Nature *457*, 277–280.

24. Karumbayaram, S., Kelly, T.K., Paucar, A.A., Roe, A.J., Umbach, J.A., Charles, A., Goldman, S.A., Kornblum, H.I., and Wiedau-Pazos, M. (2009). Human embryonic stem cell-derived motor neurons expressing SOD1 mutants exhibit typical signs of motor neuron degeneration linked to ALS. Dis. Model. Mech. *2*, 189–195.

25. Zeng, H., Guo, M., Martins-Taylor, K., Wang, X., Zhang, Z., Park, J.W., Zhan, S., Kronenberg, M.S., Lichtler, A., Liu, H.X., et al. (2010). Specification of region-specific neurons including forebrain glutamatergic neurons from human induced pluripotent stem cells. PLoS ONE *5*, e11853.

26. Amoroso, M.W., Croft, G.F., Williams, D.J., O'Keeffe, S., Carrasco, M.A., Davis, A.R., Roybon, L., Oakley, D.H., Maniatis, T., Henderson, C.E., and Wichterle, H. (2013). Accelerated high-yield generation of limb-innervating motor neurons from human stem cells. J. Neurosci. *33*, 574–586.

27. Grunseich, C., Zukosky, K., Kats, I.R., Ghosh, L., Harmison, G.G., Bott, L.C., Rinaldi, C., Chen, K.L., Chen, G., Boehm, M., and Fischbeck, K.H. (2014). Stem cell-derived motor neurons from spinal and bulbar muscular atrophy patients. Neurobiol. Dis. *70*, 12–20.

28. Maury, Y., Côme, J., Piskorowski, R.A., Salah-Mohellibi, N., Chevaleyre, V., Peschanski, M., Martinat, C., and Nedelec, S. (2015). Combinatorial analysis of developmental cues efficiently converts human pluripotent stem cells into multiple neuronal subtypes. Nat. Biotechnol. *33*, 89–96.

29. Qu, Q., Li, D., Louis, K.R., Li, X., Yang, H., Sun, Q., Crandall, S.R., Tsang, S., Zhou, J., Cox, C.L., et al. (2014). High-efficiency motor neuron differentiation from human pluripotent stem cells and the function of Islet-1. Nat. Commun. *5*, 3449.

30. Hester, M.E., Murtha, M.J., Song, S., Rao, M., Miranda, C.J., Meyer, K., Tian, J., Boulting, G., Schaffer, D.V., Zhu, M.X., et al. (2011). Rapid and efficient generation of functional motor neurons from human pluripotent stem cells using gene delivered transcription factor codes. Mol. Ther. *19*, 1905–1912.

31. Son, E.Y., Ichida, J.K., Wainger, B.J., Toma, J.S., Rafuse, V.F., Woolf, C.J., and Eggan, K. (2011). Conversion of mouse and human fibroblasts into functional spinal motor neurons. Cell Stem Cell *9*, 205–218.

32. Mazzoni, E.O., Mahony, S., Closser, M., Morrison, C.A., Nedelec, S., Williams, D.J., An, D., Gifford, D.K., and Wichterle, H. (2013). Synergistic binding of transcription factors to cell-specific enhancers programs motor neuron identity. Nat. Neurosci. *16*, 1219–1227.

33. Thomas, C.E., Ehrhardt, A., and Kay, M.A. (2003). Progress and problems with the use of viral vectors for gene therapy. Nat. Rev. Genet. *4*, 346–358.

34. Bitzer, M., Armeanu, S., Lauer, U.M., and Neubert, W.J. (2003). Sendai virus vectors as an emerging negative-strand RNA viral vector system. J. Gene Med. *5*, 543–553.

35. Aizawa, E., Hirabayashi, Y., Iwanaga, Y., Suzuki, K., Sakurai, K., Shimoji, M., Aiba, K., Wada, T., Tooi, N., Kawase, E., et al. (2012). Efficient and accurate homologous recombination in hESCs and hiPSCs using helper-dependent adenoviral vectors. Mol. Ther. *20*, 424–431.

36. Patani, R., Hollins, A.J., Wishart, T.M., Puddifoot, C.A., Alvarez, S., de Lera, A.R., Wyllie, D.J., Compston, D.A., Pedersen, R.A., Gillingwater, T.H., et al. (2011). Retinoid-independent motor neurogenesis from human embryonic stem cells reveals a medial columnar ground state. Nat. Commun. *2*, 214.

37. Gurney, M.E., Pu, H., Chiu, A.Y., Dal Canto, M.C., Polchow, C.Y., Alexander, D.D., Caliendo, J., Hentati, A., Kwon, Y.W., Deng, H.X., et al. (1994). Motor neuron degeneration in mice that express a human Cu,Zn superoxide dismutase mutation. Science *264*, 1772–1775.

38. Wegorzewska, I., Bell, S., Cairns, N.J., Miller, T.M., and Baloh, R.H. (2009). TDP-43 mutant transgenic mice develop features of ALS and frontotemporal lobar degeneration. Proc. Natl. Acad. Sci. USA *106*, 18809–18814.

39. Okita, K., Ichisaka, T., and Yamanaka, S. (2007). Generation of germline-competent induced pluripotent stem cells. Nature *448*, 313–317.

40. Takahashi, K., Okita, K., Nakagawa, M., and Yamanaka, S. (2007). Induction of pluripotent stem cells from fibroblast cultures. Nat. Protoc. *2*, 3081–3089.

41. Hatzipetros, T., Bogdanik, L.P., Tassinari, V.R., Kidd, J.D., Moreno, A.J., Davis, C., Osborne, M., Austin, A., Vieira, F.G., Lutz, C., and Perrin, S. (2014). C57BL/6J congenic Prp-TDP43A315T mice develop progressive neurodegeneration in the myenteric plexus of the colon without exhibiting key features of ALS. Brain Res. *1584*, 59–72.

42. Li, H.O., Zhu, Y.F., Asakawa, M., Kuma, H., Hirata, T., Ueda, Y., Lee, Y.S., Fukumura, M., Iida, A., Kato, A., et al. (2000). A cytoplasmic RNA vector derived from nontransmissible Sendai virus with efficient gene transfer and expression. J. Virol. *74*, 6564–6569.

43. Masaki, I., Yonemitsu, Y., Komori, K., Ueno, H., Nakashima, Y., Nakagawa, K., Fukumura, M., Kato, A., Hasan, M.K., Nagai, Y., et al. (2001). Recombinant Sendai virus-mediated gene transfer to vasculature: a new class of efficient gene transfer vector to the vascular system. FASEB J. *15*, 1294–1296.

44. Hashimoto, N., Kiyono, T., Wada, M.R., Shimizu, S., Yasumoto, S., and Inagawa, M. (2006). Immortalization of human myogenic progenitor cell clone retaining multipotentiality. Biochem. Biophys. Res. Commun. *348*, 1383–1388.

OMTM, Volume *4*

Supplemental Information

Simple Derivation of Spinal Motor Neurons from ESCs/iPSCs Using Sendai Virus Vectors

Kazuya Goto, Keiko Imamura, Kenichi Komatsu, Kohnosuke Mitani, Kazuhiro Aiba, Norio Nakatsuji, Makoto Inoue, Akihiro Kawata, Hirofumi Yamashita, Ryosuke Takahashi, and Haruhisa Inoue

Supplemental Information

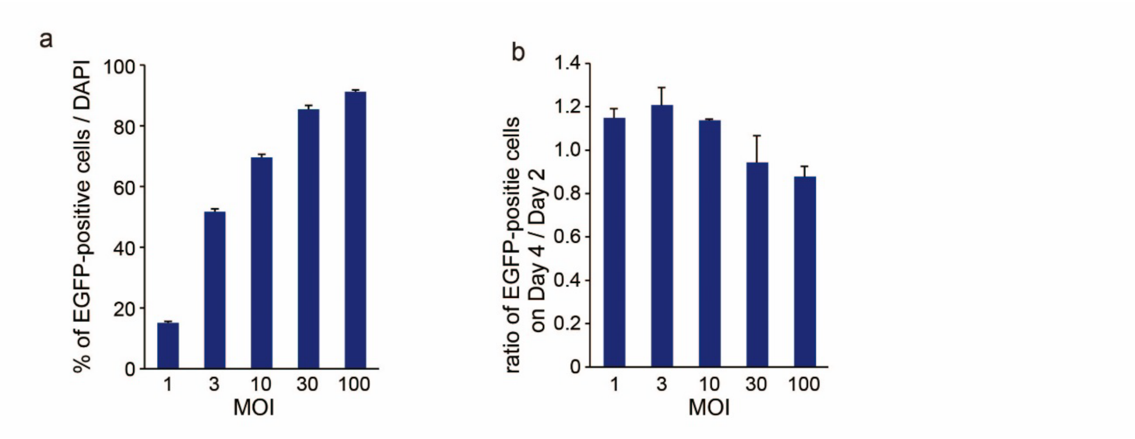

Fig. S1. Transduction of SeV-EGFP vector into iPSCs.

(a) Transduction efficiency of SeV-EGFP into iPSCs. Percentages of EGFP-positive cells on Day 2 were 15.1 ± 0.5%, 51.7 ± 1.0%, 69.6 ± 1.1%, 85.4 ± 1.3%, and 91.2 ± 0.6% at MOIs of 1, 3, 10, 30, and 100, respectively. Error bars are SEM, n = 3. (b) Ratios of EGFP-positive cells on Day 4 to Day 2 were 1.15 ± 0.04, 1.21 ± 0.08, 1.14 ± 0.01, 0.94 ± 0.12, and 0.88 ± 0.05 at MOIs of 1, 3, 10, 30, and 100, respectively. Error bars are SEM, n = 3.

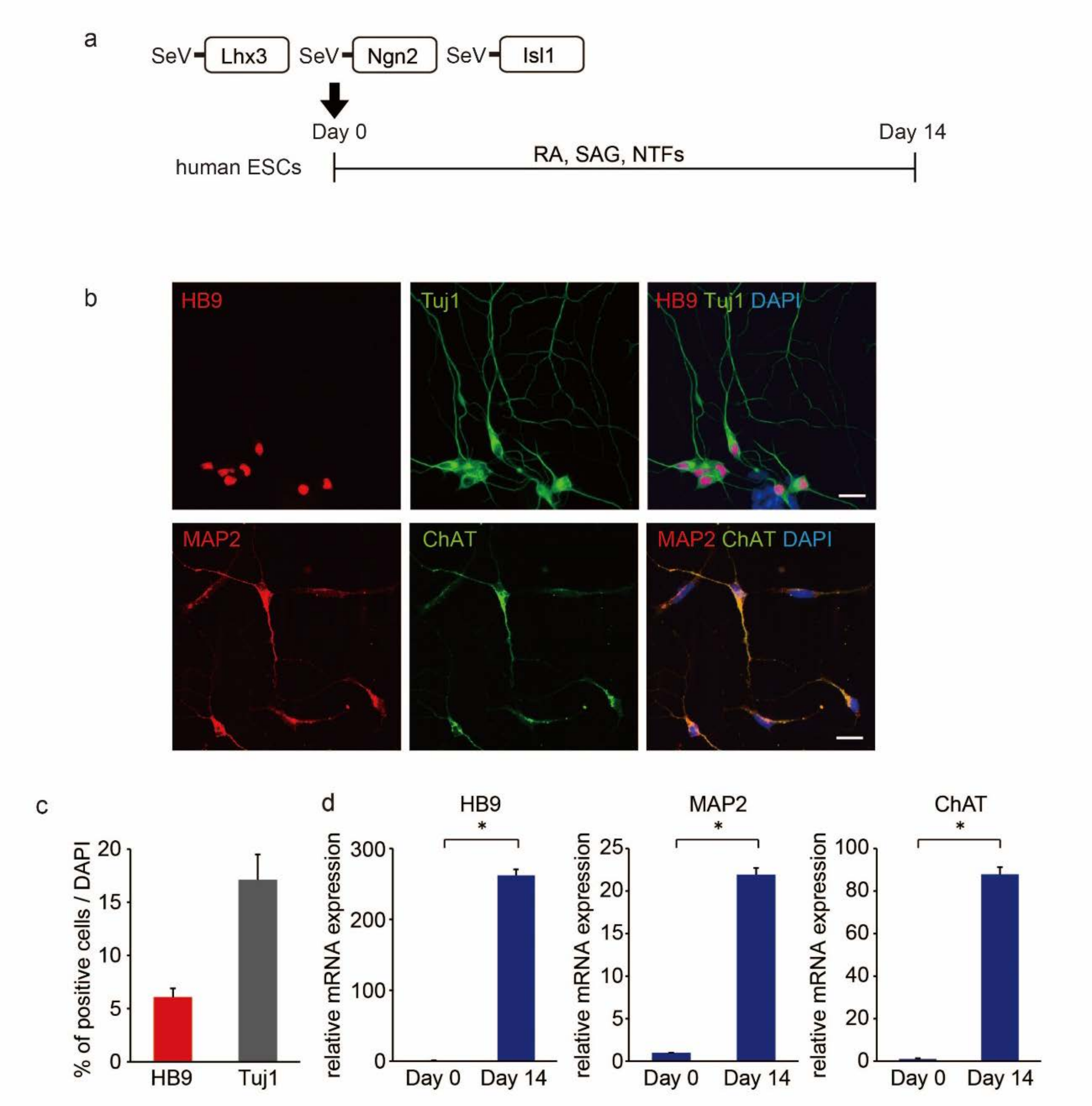

Figure S2. Differentiation of MNs from ESCs with three separate Sendai virus vectors.

(a) Outline of the experimental protocol to generate MNs using the single vectors SeV-L, SeV-N, and SeV-I in H9 ESCs. (b) Immunofluorescence staining showed the expression of MN markers (HB9 and ChAT) and neuronal markers (Tuj1 and MAP2). Scale bars, 20 µm. (c) Differentiation efficiency of MNs in SeV-infected cells. The percentages of HB9-positive and Tuj1-positive cells per total cells on Day 14 were 6.1 ± 0.8% and 17.1 ± 2.4%, respectively. (d) The qPCR analysis

of differentiated cells on Days 0 and 14 for MN markers (HB9 and ChAT) and neuronal marker (MAP2). Student's t-test was used for statistical comparison. *$p < 0.05$ versus Day 0. Error bars are SEM, $n = 3$.

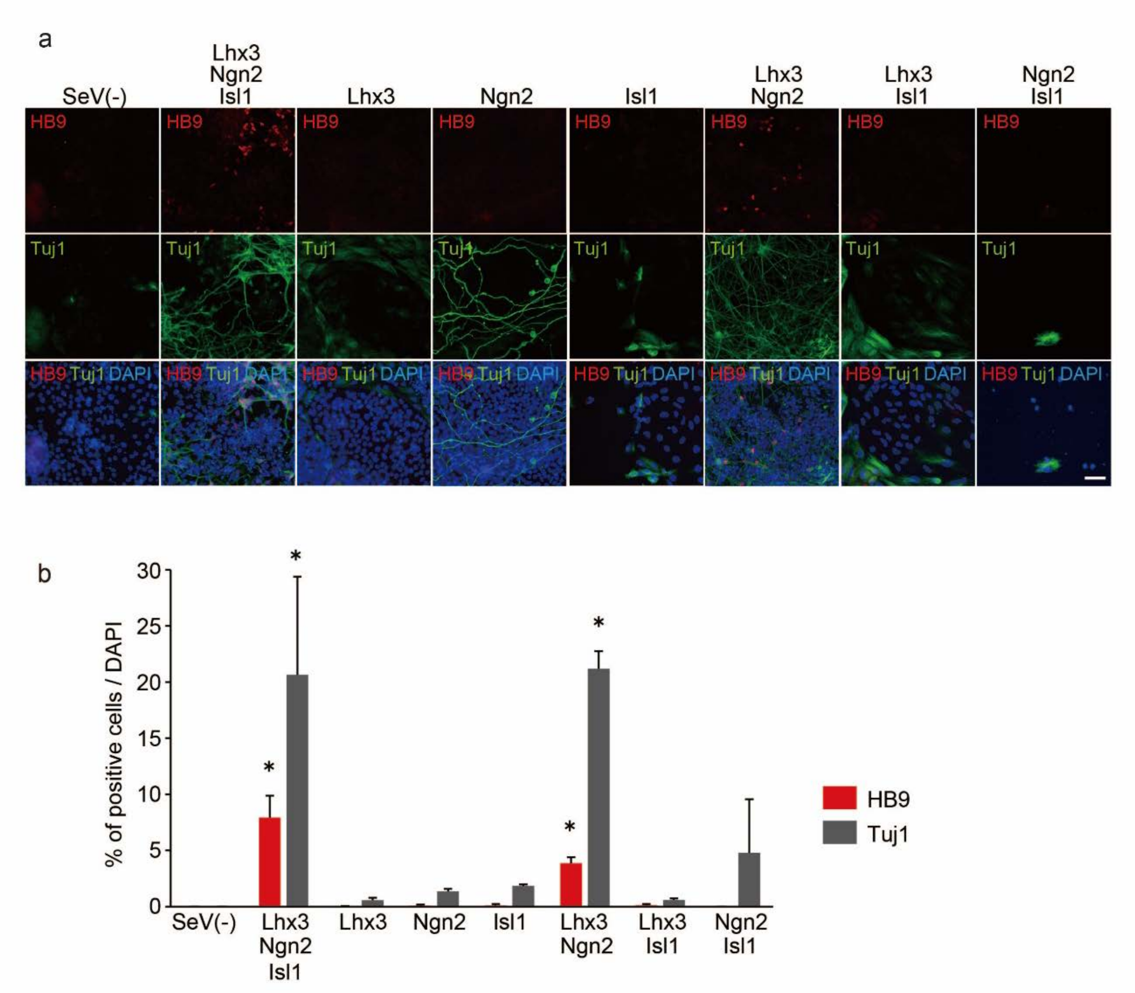

Figure S3. Comparison of combinations of three SeV vectors.

(a) Immunofluorescence staining of cells differentiated by various combinations of SeV-Lhx3 (Lhx3), SeV-Ngn2 (Ngn2), and SeV-Isl1 (Isl1) for HB9 (*top*) and Tuj1(*middle*); bottom row shows a merging of these images along with DAPI-stained nuclei (*blue*). Scale bar, 40 µm. (b) Percentages of HB9- (MNs; red) and Tuj1- (neurons; gray) positive cells derived by various combinations of the transcription factors. Data were analyzed by one-way ANOVA and Dunnett's post-hoc analysis. *$p < 0.05$. Error bars are SEM, n = 3.

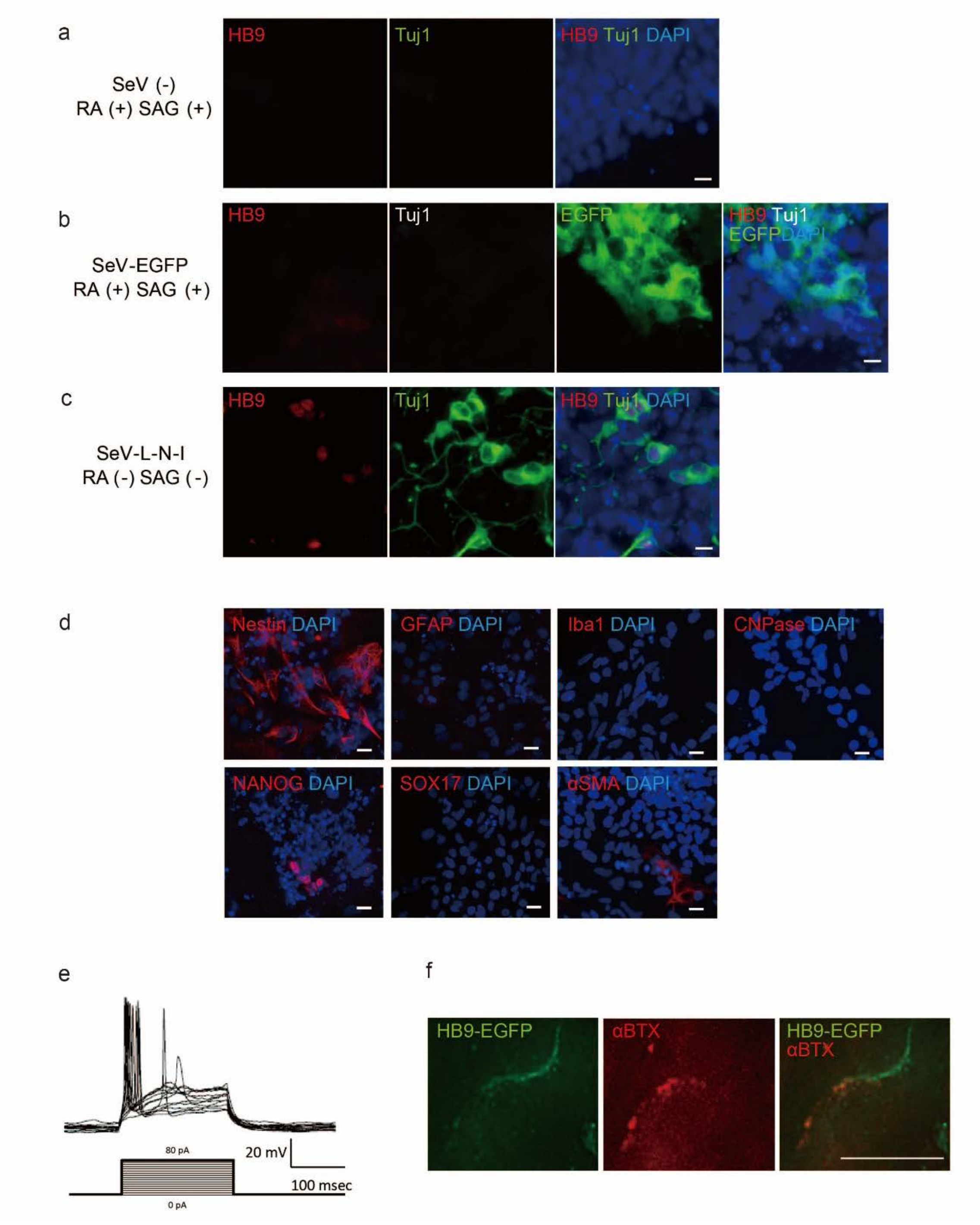

Fig. S4. Further analysis of cell culture by a single SeV vector encoding Lhx3, Ngn2, and Isl1.

(a) Differentiation of iPSCs using the current protocol without SeV vector. Tuj1- or HB9-positive cells were not observed. Scale bar, 20 µm. (b) Differentiation of iPSCs using the current protocol with SeV-EGFP vector. Tuj1- or HB9-positive cells were not observed. Scale bar, 20 µm. (c) Immunostaining of cells with SeV-L-N-I without RA and SAG. HB9-positive cells were 48.5 ± 1.5% of neurons. Scale bar, 20 µm. (d) Immunostaining for Nestin, GFAP, Iba1, CNPase, NANOG, SOX17, and αSMA on Day 14. The majority of non-neural cells were positive for Nestin. The number of Nestin-positive, NANOG-positive, and αSMA-positive cells in DAPI-stained total cells were 67.8 ± 2.7%, 0.49 ± 0.26%, and 0.27 ± 0.11%, respectively. Stainings for GFAP, SOX17, Iba1, and CNPase were negative. Scale bars, 20 µm. (e) Current-clamp recordings of action potentials on Day 21. (f) Double immunostaining against GFP for MNs and α-bungarotoxin for acetylcholine receptors on Day 21. Scale bar, 10 µm.

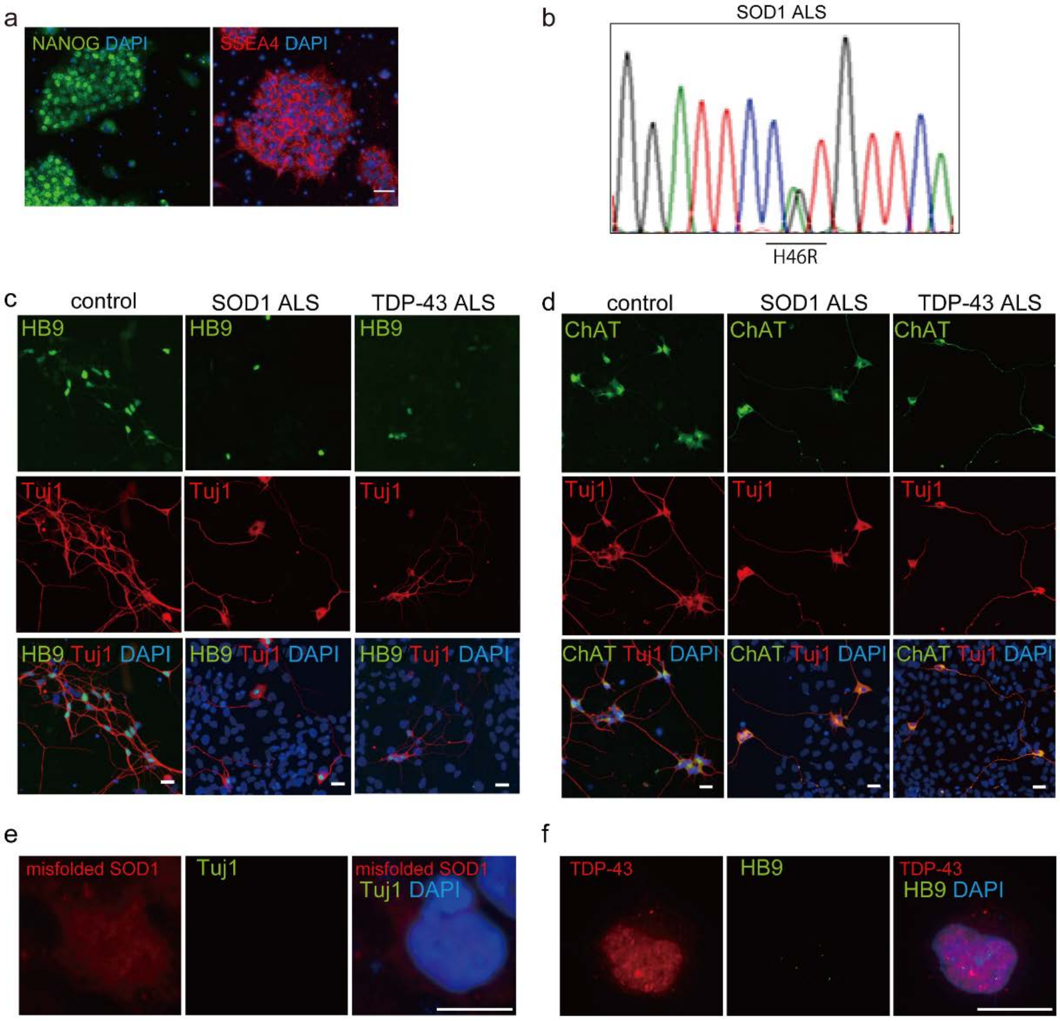

Figure S5. Differentiation of MNs from human control, SOD1-ALS and TDP-43-ALS iPSCs using a single SeV vector encoding Lhx3, Ngn2, and Isl1

(a) Immunofluorescence staining for NANOG and SSEA4 in human iPSCs derived from an SOD1-ALS patient. Scale bar, 30 µm. (b) Genomic DNA sequences for SOD1 mutation in human SOD1-ALS iPSCs. (c) Immunofluorescence staining of Tuj1 and HB9 in cells induced *via* SeV-L-N-I. Scale bars, 20 µm. (d) Immunofluorescence staining of Tuj1 and ChAT in cells induced *via* SeV-L-N-I. Scale bars, 20 µm. (e) SOD1-ALS iPSC-derived non-MNs were immunostained

with misfolded SOD1 and Tuj1 antibodies. Scale bar, 10 μm. (f) TDP-43-ALS iPSC-derived non-MNs were immunostained with TDP-43 and HB9 antibodies. Scale bar, 10 μm.

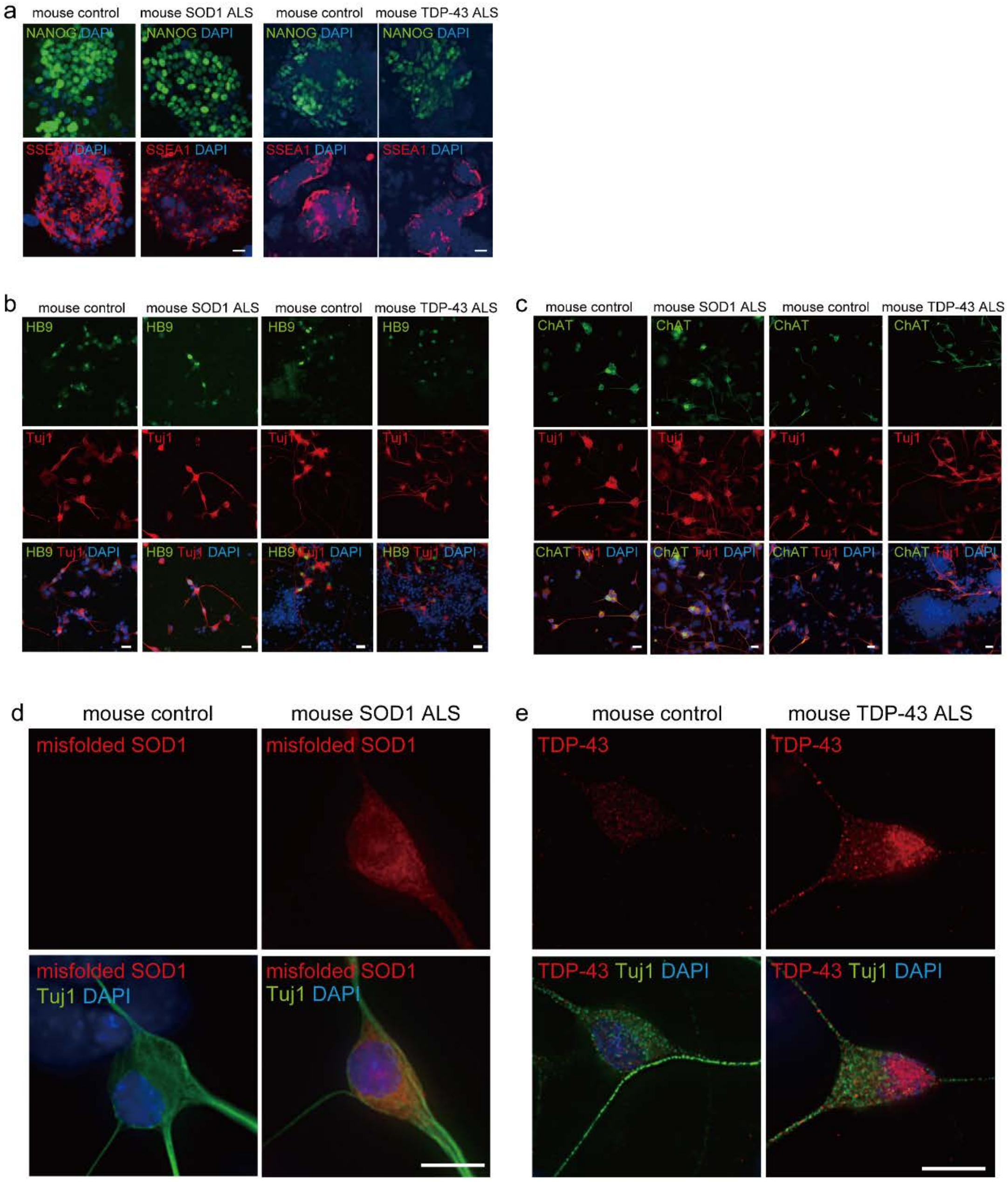

Figure S6. Phenotypes of SOD1-ALS and TDP-43-ALS mouse iPSC-derived neurons by a single SeV vector encoding Lhx3, Ngn2, and Isl1. (a) Immunofluorescence staining for NANOG and SSEA1 in control, SOD1-ALS and TDP-43-ALS mouse iPSCs. Scale bar, 30 µm. (b) Immunofluorescence staining of Tuj1 and HB9 in cells induced *via* SeV-L-N-I. Scale bars, 20 µm. (c) Immunofluorescence staining of Tuj1 and ChAT in cells induced *via* SeV-L-N-I.

Scale bar, 20 µm. (d) Control and SOD1-ALS mouse iPSC-derived MNs immunostained for misfolded SOD1 and Tuj1. Scale bar, 10 µm. (e) Control and TDP-43-ALS mouse iPSC-derived MNs were immunostained for TDP-43 and Tuj1. Scale bar, 10 µm.

Table S1. MN differentiation from iPSCs using signaling molecules.

Reference	Compounds for MN induction	Efficiency of MN differentiation	Days from iPSCs to MNs
Dimos et al., 2008	RA, SHH agonist	20% (HB9+ / all cells)	38 days
Ebert et al., 2009	RA, SHH	12.6 ± 2.2%, 9.5 ± 2.4% (ChAT+ and Tuj1+ / total cells)	28 days
Karumbayaram et al., 2009	RA, SHH	28.2 ± 5.7%, 33.6 ± 12% (ISL1+ / Tuj1+)	35-49 days
Zeng et al., 2010	RA, SHH	～20% (HB9+ / total cells)	35 days
Mitne-Neto et al., 2011	RA, SHH	～5% (HB9+ / total cells)	49 days
Amoroso et al., 2013	SB431542, LDN193189, RA, SAG, PUR	27 ± 1% (HB9+ or ISL1+ / DAPI)	21 days
Sareen et al., 2013	ATRA, purmorphamine	33-45% (SMI32+ / total cells)	49 days
Qu et al., 2014	Compound C, RA, SHH,	～2%, 51.6 ± 5.7% (HB9+ / DAPI)	18 days
Grunseich et al., 2014	SB431542, LDN193189, RA, SAG, PUR	20-30% (HB9+ / total cells)	21-30 days
Maury et al., 2015	SB431542, LDN193189, SAG, RA,CHIR99021, FGF2, DAPT	74% (HB9+ and ISL1+ / neurons)	14 days
Du et al., 2015	SB431542, DMH1, PUR, RA,CHIR99021, CpdE	91 ± 6% (ChAT+ / neurons)	28 days

Abbreviations: RA, retinoic acid; SHH, sonic hedgehog; SAG, smoothened agonist; PUR, purmorphamine; ATRA, all-trans retinoic acid; FGF2, fibroblast growth factor 2; DAPT, N-[N-(3,5-difluorophenacetyl)-l-alanyl]-S-phenylglycine t-butyl ester; Cpd E, Compound E.

Table S2. MN differentiation from ESCs/iPSCs or fibroblasts using transcription factors (TFs).

Reference	Initial source	TFs for MN induction	Efficiency of MN differentiation	HB9 or Phox2b detected days from ESCs/iPSCs	Medium
Hester et al., 2011	Human ESCs/iPSC	Ngn2, Isl1, Lhx3	55% (HB9+ / DAPI)	21 days	3 types
Son et al., 2011	HEF	Ascl1, Brn2, Myt1l, Lhx3,Hb9, Isl1, Ngn2, NeuroD1	～0.01% (Hb9+ / HEF)	30 days	2 types
Son et al., 2011	MEF	Ascl1, Brn2, Myt1l, Lhx3, Hb9, Isl1, Ngn2	5-10% (Hb9+ / MEF)	5 days	2 types
Mazzioni et al., 2013	Mouse ESC	Ngn2, Isl1, Lhx3	99.82 ± 0.17 (Hb9+ / transgenic cells)	1 day	2 types
Mazzioni et al., 2013	Mouse ESC	Ngn2, Isl1, Phox2a	99.03% (Phox2b+ / transgenic cells	3 days	2 types
This study	Human iPSC	Ngn2, Isl1, Lhx3	92.8 ± 1.2% (HB9+ / infected cells)	2 days	1 type

Abbreviations: ESC, embryonic stem cell; HEF, human embryonic fibroblast; MEF, mouse embryonic fibroblast.

Table S3. Comparison of virus vectors

Vector	Genetic material	Vector genome forms	Tropism	Packaging capacity
Retrovirus	RNA	Integrated	Dividing cells	～8kb
Lentivirus	RNA	Integrated	Broad	～8kb
Adenovirus	DNA	Episomal	Broad	～8-30kb
AAV	DNA	Episomal (90%)<br>Integrated (10%)	Broad	～5kb
Sendai virus	RNA	Episomal	Broad	～5kb

Abbreviations: AAV, adeno-associated virus

Table S4. List of human iPSC clones

human iPSC clone				
Clone name in this study	HB9-EGFP knockin	control	SOD1 ALS	TDP-43 ALS
Clone name at establishment	24-13	201B7	A30-1-1-1	A3411
Sex	Male	Female	Female	Female
Donor Age	New born	38	51	62
Origin	Fibroblast	Fibroblast	Fibroblast	Fibroblast
Reprogramming vector	Retrovirus	Retrovirus	Episomal	Episomal
Mutation	−	−	SOD1 (H46R)	TDP-43 (M337V)

Table S5. List of mouse iPSC clones

mouse iPSC clone				
Clone name in this study	control (SOD1)	mouse SOD1 ALS	control (TDP-43)	mouse TDP-43 ALS
Clone name at establishment	N-4	G-10	M7-5	M5-3
Origin	MEF	MEF	MEF	MEF
Reprogramming vector	Retrovirus	Retrovirus	Retrovirus	Retrovirus
Mutation	−	SOD1 (G93A)	−	TDP-43 (A315T)

Table S6. List of primers

Primers	Sequences (5' to 3')	Applications
HB9-Fw	tgcctaagatgcccgactt	qPCR
HB9-Rv	agctgctggctggtgaag	qPCR
ChAT-Fw	tgaaacctacctgatgagcaac	qPCR
ChAT-Rv	agcagaacatctccgtggtt	qPCR
MAP2-Fw	caggtggcggacgtgtgaaaattgagagtg	qPCR
MAP2-Rv	cacgctggatctgcctggggactgtg	qPCR
HOXB4-Fw	ctggatgcgcaaagttcac	qPCR
HOXB4-Rv	ttccttctccagctccaaga	qPCR
HOXC6-Fw	ccaggaccagaaagccagta	qPCR
HOXC6-Rv	ggtaccgcgagtagatctgg	qPCR
HOXC9-Fw	cagcaagcacaaagaggaga	qPCR
HOXC9-Rv	tccagcgtctggtacttgg	qPCR
HOXC10-Fw	gacacctcggataacgaagc	qPCR
HOXC10-Rv	cctcttcttccttccgctct	qPCR
GAPDH-Fw	tccactggcgtcttcacc	qPCR
GAPDH-Rv	ggcagagatgatgaccctttt	qPCR

Movie S1. Time-lapse imaging of EGFP-positive cells induced with a single SeV vector encoding Lhx3, Ngn2, and Isl1 to HB9-EGFP knock-in human iPSCs.

Time-lapse imaging was conducted from Day 1. The images were captured every 30 minutes. Recording duration was 50 hours.
